# Differential patterns of reproductive and lifestyle risk factors for breast cancer according to birth cohorts among women in China, Japan and Korea

**DOI:** 10.1186/s13058-024-01766-0

**Published:** 2024-01-22

**Authors:** Salma Nabila, Ji-Yeob Choi, Sarah Krull Abe, Md Rashedul Islam, Md Shafiur Rahman, Eiko Saito, Aesun Shin, Melissa A. Merritt, Ryoko Katagiri, Xiao-Ou Shu, Norie Sawada, Akiko Tamakoshi, Ritsu Sakata, Atsushi Hozawa, Jeongseon Kim, Chisato Nagata, Sue K. Park, Sun-Seog Kweon, Hui Cai, Shoichiro Tsugane, Takashi Kimura, Seiki Kanemura, Yumi Sugawara, Keiko Wada, Min-Ho Shin, Habibul Ahsan, Paolo Boffetta, Kee Seng Chia, Keitaro Matsuo, You-Lin Qiao, Nathaniel Rothman, Wei Zheng, Manami Inoue, Daehee Kang

**Affiliations:** 1 Department of Biomedical Sciences, Seoul National University Graduate School, 103 Daehak-ro, Jongno-gu, 03080 Seoul, Republic of Korea; 2 BK21plus Biomedical Science Project, Seoul National University College of Medicine, Seoul, Republic of Korea; 3 Institute of Health Policy and Management, Seoul National University Medical Research Center, Seoul, Republic of Korea; 4 Cancer Research Institute, Seoul National University, Seoul, Republic of Korea; 5 Division of Prevention, National Cancer Center Institute for Cancer Control, Tokyo, Japan; 6 Hitotsubashi Institute for Advanced Study, Hitotsubashi University, Tokyo, Japan; 7 Research Center for Child Mental Development, Hamamatsu University School of Medicine, Hamamatsu, Japan; 8 National Center for Global Health and Medicine, Institute for Global Health Policy Research, Tokyo, Japan; 9 Department of Preventive Medicine, Seoul National University College of Medicine, Seoul, Republic of Korea; 10 The Daffodil Centre, The University of Sydney, A Joint Venture with Cancer Council NSW, Sydney, Australia; 11 Division of Cohort Research, National Cancer Center Institute for Cancer Control, Tokyo, Japan; 12 National Institute of Health and Nutrition, National Institutes of Biomedical Innovation, Health and Nutrition, Tokyo, Japan; 13 Division of Epidemiology, Vanderbilt-Ingram Cancer Center, Vanderbilt Epidemiology Center, Vanderbilt University Medical Center, Nashville, TN USA; 14 Department of Public Health, Faculty of Medicine, Hokkaido University, Sapporo, Japan; 15 Radiation Effects Research Foundation, Hiroshima, Japan; 16 Tohoku University Graduate School of Medicine, Sendai, Miyagi Prefecture Japan; 17 Graduate School of Cancer Science and Policy, National Cancer Center, Goyang-si, Gyeonggi-do Republic of Korea; 18 Department of Epidemiology and Preventive Medicine, Gifu University Graduate School of Medicine, Gifu, Japan; 19 Department of Preventive Medicine, Chonnam National University Medical School, Gwangju, Republic of Korea; 20 Department of Public Health Sciences, University of Chicago, Chicago, IL USA; 21 Stony Brook Cancer Center, Stony Brook University, Stony Brook, NY USA; 22 Department of Medical and Surgical Sciences, University of Bologna, Bologna, Italy; 23 Saw Swee Hock School of Public Health, National University of Singapore, Singapore, Singapore; 24 Division Cancer Epidemiology and Prevention, Aichi Cancer Center Research Institute, Nagoya, Japan; 25 Department of Cancer Epidemiology, Nagoya University Graduate School of Medicine, Nagoya, Japan; 26 School of Population Medicine and Public Health, Chinese Academy of Medical Sciences and Peking Union Medical College, Beijing, China; 27 Division of Cancer Epidemiology and Genetics, Occupational and Environmental Epidemiology Branch, National Cancer Institute, Bethesda, MD USA

**Keywords:** Breast cancer, Birth cohort, Reproductive factors, Lifestyle factors, Women, Asia, Background

## Abstract

**Background:**

The birth cohort effect has been suggested to influence the rate of breast cancer incidence and the trends of associated reproductive and lifestyle factors. We conducted a cohort study to determine whether a differential pattern of associations exists between certain factors and breast cancer risk based on birth cohorts.

**Methods:**

This was a cohort study using pooled data from 12 cohort studies. We analysed associations between reproductive (menarche age, menopause age, parity and age at first delivery) and lifestyle (smoking and alcohol consumption) factors and breast cancer risk. We obtained hazard ratios (HRs) with 95% confidence intervals (CIs) using the Cox proportional hazard regression analysis on the 1920s, 1930s, 1940s and 1950s birth cohorts.

**Results:**

Parity was found to lower the risk of breast cancer in the older but not in the younger birth cohort, whereas lifestyle factors showed associations with breast cancer risk only among the participants born in the 1950s. In the younger birth cohort group, the effect size was lower for parous women compared to the other cohort groups (HR [95% CI] 0.86 [0.66–1.13] compared to 0.60 [0.49–0.73], 0.46 [0.38–0.56] and 0.62 [0.51–0.77]). Meanwhile, a higher effect size was found for smoking (1.45 [1.14–1.84] compared to 1.25 [0.99–1.58], 1.06 [0.85–1.32] and 0.86 [0.69–1.08]) and alcohol consumption (1.22 [1.01–1.48] compared to 1.10 [0.90–1.33], 1.15 [0.96–1.38], and 1.07 [0.91–1.26]).

**Conclusion:**

We observed different associations of parity, smoking and alcohol consumption with breast cancer risk across various birth cohorts.

**Supplementary Information:**

The online version contains supplementary material available at 10.1186/s13058-024-01766-0.

## Key messages

- There are associations between reproductive factors and breast cancer risk.
- There are associations between smoking and alcohol consumption and increased breast cancer risk among Asian women.
- There are differential patterns of parity, age at first delivery and smoking status across birth cohorts.
- Reproductive risk factors were more apparent in the older birth cohorts, whereas smoking and alcohol consumption were only notable in the younger generation.

**Supplementary Information:**

The online version contains supplementary material available at 10.1186/s13058-024-01766-0.

Breast cancer is reportedly the most commonly diagnosed type of cancer worldwide and the leading cause of cancer mortality among women [1, 2]. Although the incidence rate in Asia has been rather low historically, recent trends showed that it is rapidly increasing in several Asian countries, including Japan, China and Korea, with the rates now approaching 76.3, 39.1 and 64.2 (per 100,000 women), respectively, in these countries [3, 4]. The change in secular breast cancer trends in Asia has been attributed to birth cohort effects by some previous studies [5–8].

Reproductive factors, including menarche age, parity, age at first delivery and menopause age, and lifestyle factors, such as smoking, are widely known as important considerations in the assessment of breast cancer risk [9]. Similar to those of breast cancer, secular trends of reproductive and lifestyle factors have also been observed in previous studies, for example, a decrease in menarche age [10, 11], an increase in menopause age [12, 13], a decrease in parity number [12] and an increase in the proportion of women who smoke [10]. Given that both breast cancer incidence and its determinants have been suggested to show notable trends based on birth cohorts, the associations between certain factors and breast cancer risk may also differ according to the birth cohort.

Therefore, we conducted a cohort study to determine whether a difference exists in the associations between reproductive and lifestyle factors and breast cancer risk according to birth cohorts using a large sample of the Asian population from 12 prospective cohort studies from China, Japan and Korea.

## Methods

### Study design and population

This cohort study was conducted based on the Asia Cohort Consortium (ACC), an international collaborative project to combine existing cohorts across Asia that currently involves more than a million participants. A more detailed description of the ACC has been given in previous articles [14]. Among all the participating cohorts in the ACC, 12 cohorts that had agreed to participate in this study were included. These cohorts were from China, Japan and Korea, comprising the following studies: Shanghai Women’s Health Study (SWHS) [15], Japan Public Health Centre-based Prospective Study I and II (JPHC I and JPHC II) [16, 17], Japan Collaborative Cohort Study (JACC) [18], Life Span Study Cohort (LSS) [19], Miyagi Cohort Study (Miyagi) [20], Ohsaki National Health Insurance Cohort Study (Ohsaki) [20], Korean National Cancer Centre Cohort (KNCC) [21], Takayama Study [22], Three Prefecture Cohort Study Miyagi (3 Pref. Miyagi) [23], Korean Multi-center Cancer Cohort Study (KMCC) [24], and The Namwon Study (Namwon) [25].

All data harmonisation was performed centrally by the ACC coordinating centre. The centre sent the details of the data processing method to each cohort so that the data could be processed following the same method and then sent back to the coordinating centre. After verifying the data content, the ACC coordinating centre organised and pooled all the data. After signing an agreement form, investigators could access and analyse the data using remote access to a virtual private network or the computer at the National Cancer Center in Tokyo, Japan. A working group was created to process and derive the necessary reproductive variables to ensure that investigators had standardised variables and used the data appropriately. Detailed information on the cleaning of the reproductive variables by the working group has been given in another article [26].

**Fig. 1:**
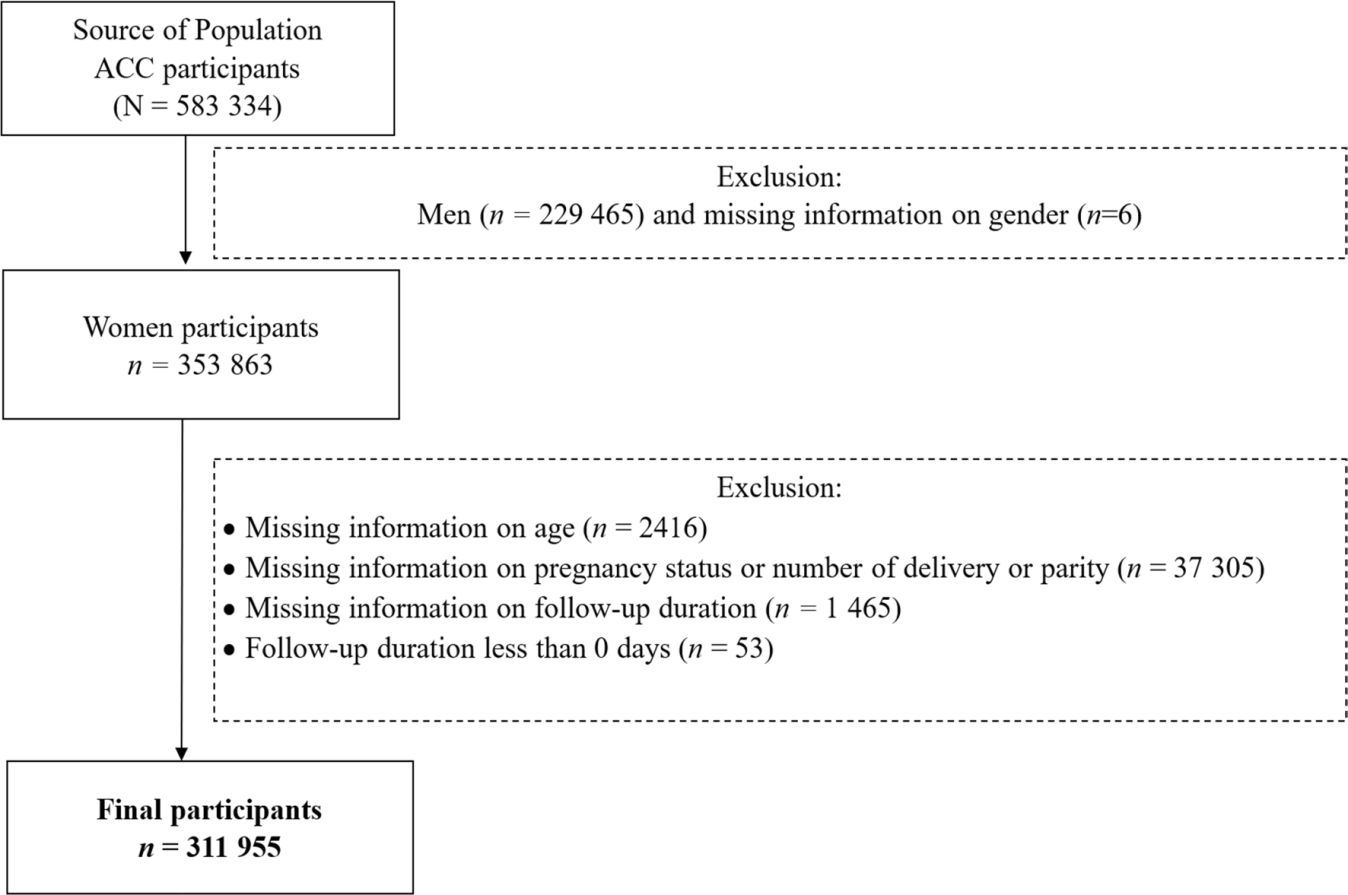
Participants selection

Of the 583,334 participants from the 12 cohorts, men (n = 229,465) and participants with missing information on sex (n = 6) were excluded; thus, 353,863 women remained. Further exclusions were made for participants with missing information on age (n = 2416), pregnancy status or number of deliveries or parity (n = 37 305), follow-up duration (n = 1465), as well as those with a follow-up duration of less than 0 days (n = 53). Finally, 311,955 participants were included in this study, including 4581 breast cancer cases. A flow chart of participant selection is presented in Fig. 1, and the details of participant selection from each cohort study are provided in Additional file 1: Table S1. Although a large number of participants were excluded because of missing information, most of the basic characteristics did not show large differences between excluded and included participants in the evaluation of the standardised differences (absolute value < 0.5), except for the number of children (Additional file 1: Table S2) [27].

The pooled analysis of the ACC cohorts was approved by the ethics committee of the National Cancer Centre Japan (number 2014-041), and each participating study was approved by the respective overseeing ethics committee. Informed consent was previously collected from the participants before each cohort study. All methods included in the present study were performed in accordance with relevant guidelines and regulations.

### Exposure and outcome

The exposure in this study consisted of reproductive factors (menarche age, parity, age at first delivery and menopause age) and lifestyle factors (smoking and alcohol consumption), which were collected by self-report questionnaire in each cohort study. Menarche age was grouped into < 13, 13–14, 15–16 and ≥ 17 years; parity was grouped into nulliparous and parous; age at first delivery was grouped into ≤ 20, 21–25, 26–30, > 30 years and nulliparous; and menopause age was grouped into < 45, 45–49, 50–54 and ≥ 55 years. Smoking status was defined as never and ever smokers and alcohol consumption was defined as non-current drinkers and current drinkers, with non-current drinkers comprising both participants who never drank and past drinkers.

The reproductive working group in the ACC was responsible for harmonising and cleaning the reproductive variables. Missing data for menopausal status was inputted as post-menopause if there was information on the age of menopause or if the baseline age was more than 54 years, as pre-menopause if the baseline age was less than 44 years, and as missing or unknown if the baseline age was 45–53 years. Furthermore, the plausible range of age at menarche was defined as 10–23 years, menopause age as 20 years or more, and age at first delivery as 10–49 years. Responses outside these ranges were considered implausible and thus treated as missing.

Breast cancer cases were defined with reference to the International Classification of Diseases for Oncology as codes C50.0–50.9 by each cohort study through linkages with the cancer registries. Most of the cancer registries from the participating countries meet the inclusion criteria of high-quality cancer registries and have been published in the Cancer Incidence in Five Continents by The International Agency for Research on Cancer [28]. The follow-up duration was defined as the interval between the entry date and the breast cancer incidence date for cases or the last follow-up date for non-cases.

### Statistical analysis

Cox proportional hazard regression analysis was performed to obtain the hazard ratio (HR) with a 95% confidence interval (CI) for the overall breast cancer risk from the pooled data by adjusting for baseline age and age at first delivery in model 1 and baseline age, age at first delivery and cohort studies in model 2. Considering the potential heterogeneity and effects of each study’s quality and size, a meta-analysis of the dataset was performed to assess whether the results from both analyses were comparable. Heterogeneity was evaluated using the Cochran Q and Higgins I^2^ tests; *P* < 0.1 or I2 < 50% indicated a significant difference [29].

A stratified analysis according to the birth cohort from the pooled data was performed by categorising the participants’ birth year into four groups: 1920s or earlier, 1930s, 1940s and 1950s or later. The HR for breast cancer risk in each birth year group was adjusted for baseline age, age at first delivery and cohort. The birth cohort groups in the participating cohort studies were not equally distributed; thus, the pooled analysis results might be weighted toward particular cohort studies. Therefore, as for the overall analysis, a meta-analysis was performed to determine whether the obtained risks in each birth cohort were consistent with the results of the stratified analysis of the pooled data. Additionally, the standardised difference between the included and excluded participants was assessed and stratified analyses by menopausal status and country were performed. The analysis of alcohol consumption only included 11 cohorts and excluded one cohort (Takayama Study) due to data unavailability. All analyses were performed using Stata version 16.0 software (Stata Corporation, College Station, TX).

## Results

### Overall pooled analysis

Among 311,955 participants (mean [standard deviation] age 54.2 [10.7] years), 4581 breast cancer cases were confirmed during a mean of 16.5 years of follow-up, and the mean age at diagnosis was 61.9 years (Table 1). Details of the distributions of baseline characteristics in each cohort are presented in Additional file 1: Table S3. Table 2 shows that there was an increased risk of breast cancer among participants with a younger menarche age (HR [95% CI] 1.17 [1.06–1.29] for 15–16 years, 1.34 [1.22–1.48] for 13–14 years and 1.41 [1.23–1.61] for < 13 years compared to those > 17 years in model 2). Compared to the group aged 50–54 years, a younger menopause age was associated with a decreased risk of breast cancer (0.84 [0.76–0.93] for 45–49 years and 0.82 [0.72–0.94] for < 45 years). Furthermore, breast cancer risk was lower among parous women than nulliparous women (0.61 [0.55–0.68]). Compared to the group aged 21–25 years at first delivery, the younger age group showed a lower risk of breast cancer (0.79 [0.69–0.90]); meanwhile, the older groups and nulliparous group showed a higher risk of breast cancer (1.24 [1.15–1.33] for 26–30 years, 1.52 [1.37–1.70] for > 30 years and 1.76 [1.58–1.97] for nulliparous). Participants who were ever smokers (1.13 [1.01–1.26]) and those who were current alcohol drinkers at baseline (1.15 [1.05–1.26]) also showed a higher risk of breast cancer.

**Table 1:** Baseline characteristics of participants based on birth cohort group

	Total N (%)	≤ 1920s N (%)	1930s N (%)	1940s N (%)	≥ 1950s N (%)	*P* value
	311,955	71,689 (23.0)	92,643 (29.7)	80,775 (25.9)	66,848 (21.4)	
Breast cancer cases	4581 (1.5)	707 (1.0)	1279 (1.4)	1385 (1.8)	1210 (1.8)	< 0.001
Baseline year (min–max)	1993–2015	1993–2007	1993–2013	1993–2014	1993–2015	< 0.001
Follow-up duration (years, mean ± SD)	16.5 ± 6.6	15.4 ± 8.4	17.1 ± 6.4	17.8 ± 5.8	15.4 ± 4.9	< 0.001
Follow-up duration among breast cancer cases (years, mean ± SD)	10.2 ± 6.6	11.7 ± 8.5	10.4 ± 6.6	10.4 ± 6.3	8.9 ± 5.3	< 0.001
Age at baseline (years, mean)	54.2 ± 10.7	65.3 ± 9.3	57.7 ± 7.2	48.8 ± 7.0	4401 ± 5.3	< 0.001
Age at baseline among breast cancer cases (years, mean)	51.7 ± 9.3	60.9 ± 9.8	57.3 ± 7.6	48.8 ± 6.3	44.0 ± 3.8	< 0.001
Age at diagnosis of breast cancer cases (years, mean)	61.9 ± 10.4	72.6 ± 9.1	67.6 ± 8.3	59.2 ± 7.6	52.8 ± 6.0	< 0.001
Menopausal status						< 0.001
Premenopausal	103,195 (33.1)	4206 (5.9)	7960 (8.6)	40,040 (49.6)	50,989 (76.3)	
Postmenopausal	194,390 (62.3)	66,276 (92.5)	81,975 (88.5)	35,031 (43.4)	11,108 (16.6)	
Unknown	14,370 (4.6)	1207 (1.7)	2708 (1.68)	5704 (7.6)	4751 (7.1)	
Age at menarche						< 0.001
< 13 years	19,766 (6.3)	2074 (2.9)	3451 (3.7)	7777 (9.6)	6464 (9.7)	
13–14 years	102,516 (32.9)	16,314 (22.8)	25,509 (27,5)	34,057 (42.2)	26,636 (39,9)	
15–16 years	107,179 (34.4)	26,198 (36.5)	34,547 (37.3)	24,411 (30.2)	22,023 (32.9)	
17 + years	55,673 (17.8)	16,306 (22.8)	21,726 (23.5)	9641 (11.9)	8000 (12.0)	
Missing	26,821 (8.6)	10,797 (15,1)	7410 (8,0)	4889 (6,1)	3725 (8.6)	
Age at menopause^a^						< 0.001
< 45 years	28,040 (14.4)	7633 (11.5)	10,080 (12.3)	6745 (19.3)	3582 (32.3)	
45–49 years	61,684 (31.7)	17,886 (27.0)	26,928 (32.9)	13,631 (38.9)	3239 (29.2)	
50–54 years	74,612 (38.4)	25,241 (38.1)	34,748 (42.4)	11,581 (33.1)	3042 (27.4)	
55 + years	8711 (4.5)	3588 (5.4)	3680 (4.5)	1112 (3.2)	331 (3.0)	
Missing	21,343 (11.0)	11,928 (18.0)	6539 (8.0)	1962 (5.6)	914 (8.2)	
Parity						< 0.001
Nulliparous	290,865 (93.2)	63,542 (88.6)	87,281 (94,2)	76,141 (94.3)	63,910 (96.6)	
Parous	21,090 (6.8)	8147 (11.4)	5362 (5.8)	4634 (5.7)	2947 (4.4)	
Number of children						< 0.001
0	12,601 (4.0)	3201 (4.5)	3460 (3.7)	2993 (3.7)	2947 (4.4)	
1–2	148,522 [47, 61]	13,915 (19.4)	36,444 (39.3)	44,686 (55.3)	53,477 (80.0)	
3–4	93,958 [30, 1]	23,267(32.5)	36,507 (39.4)	24,684 (30.6)	9500 (14.2)	
5 +	26,601 (8.5)	12,180 (17.0)	9934 (10.7)	3830 (4.7)	657 (1.0)	
Missing	30,273 (9.7)	19,126 (26,7)	6298 (6.8)	4582 (5.7)	267 (0.4)	
Age at first delivery (%)						< 0.001
≤ 20 years	28,979 (9.3)	9027 (12.6)	12,580 (13.6)	5582 (6.9)	1790 (2.7)	
21–25 years	146,015 (46.8)	35,408 (49.4)	47,325 (51.1)	42,575 (52.7)	20,707 (31.0)	
26–30 years	90,182 (28.9)	13,411 (18.7)	21,087 (22.8)	22,275 (27.6)	33,409 (49.98)	
> 30 years	18,275 (5.9)	3000 (4.2)	3955 (4.3)	4227 (5.2)	7093 (10.6)	
Nulliparous	21,090 (6.8)	8147 (11.4)	5362 (5.7)	2947 (4.4)	21,090 (6.8)	
Breastfeeding among parous						< 0.001
Yes	103,181 (35.5)	14,744 (23.2)	36,073 (41.3)	30,419 (40.0)	21,945 (34.3)	
No	15,828 (5.4)	860 (1.4)	4084 (4.7)	6403 (8.4)	4481 (7.0)	
Missing	171,856 (59.1)	47,938 (75.4)	47,124 (54.0)	39,319 (51,6)	37,475 (58.7)	
Smoking status						< 0.001
Never	264,230 (84.7)	53,881 (75.2)	77,561 (83.7)	70,847 (87.7)	61,941 (92.7)	
Ever	23,058 (7.4)	6842 (9.5)	6555 (7.1)	5945 (7.4)	3716 (5.6)	
Missing	24,667 (7.9)	10,966 (15.3)	8527 (9.2)	3983 (4.9)	1191 (1.8)	
Alcohol drinking						< 0.001
Non-drinker^b^	208,535 (66.9)	48,200 (67.2)	60,066 (64.8)	50,061 (62.0)	50,208 (75.1)	
Current drinker	53,682 (17.2)	11,320 (15.8)	14,304 (15.4)	16,074 (19.9)	11,984 (17.9)	
Missing	49,738 (15.9)	12,169 (17.0)	18,273 (19.7)	14,640 (18,1)	4656 (7.0)	

^a^The result regarding menopause age was based on the analysis of post-menopausal women

^b^The non-drinker category of alcohol consumption included both participants who never drank alcohol and ex-drinkers

*P* value comparisons across class categories are based on the chi-square test for categorical variables; *P* values for continuous variables are based on ANOVA

**Table 2:** Associations of reproductive factors, smoking status and alcohol consumption with breast cancer risk

	Overall		Birth cohorts					
	HR^1^ (95% CI)	HR^2^ (95% CI)	≤ 1920s HR^2^ (95% CI)	1930s HR2 (95% CI)	1940s HR2 (95% CI)	≥ 1950s HR^2^ (95% CI)	Heterogeneity Test	
							P*-*value (Q test)	I^2^
*Menarche age*								
< 13 years	1.30 (1.14–1.49)	1.41 (1.23–1.61)	1.55 (1.03–2.33)	1.54 (1.17–2.04)	1.24 (0.95–1.60)	1.30 (1.01–1.68)	0.62	0%
13–14 years	1.33 (1.20–1.46)	1.34 (1.22–1.48)	1.47 (1.17–1.84)	1.27 (1.08–1.50)	1.42 (1.16–1.75)	1.12 (0.91–1.38)	0.28	22%
15–16 years	1.19 (1.08–1.31)	1.17 (1.06–1.29)	1.07 (0.86–1.34)	1.23 (1.05–1.44)	1.18 (0.96–1.46)	1.06 (0.87–1.31)	0.63	0%
17 + years	Reference	Reference	Reference	Reference	Reference	Reference		
(cont.)	0.95 (0.93–0.96)	0.94 (0.92–0.96)	0.91 (0.86–0.95)	0.95 (0.92–0.98)	0.95 (0.92–0.98)	0.96 (0.93–1.00)	0.29	20%
*Menopause age*^*a*^								
< 45 years	0.85 (0.74–0.96)	0.82 (0.72–0.94)	0.65 (0.48–0.87)	0.98 (0.81–1.19)	0.87 (0.67–1.14)	0.92 (0.44–1.91)	0.15	43%
45–49 years	0.90 (0.82–0.99)	0.84 (0.76–0.93)	0.75 (0.61–0.92)	0.91 (0.79–1.04)	0.80 (0.66–0.98)	1.24 (0.69–2.22)	0.22	32%
50–54 years	Reference	Reference	Reference	Reference	Reference	Reference		
≥ 55 years	1.10 (0.90–1.35)	1.11 (0.91–1.35)	0.79 (0.52–1.19)	1.21 (0.93–1.59)	1.32 (0.80–2.18)	2.66 (0.98–7.21)	0.10	53%
*Parity*								
Nulliparous	Reference	Reference	Reference	Reference	Reference	Reference		
Parous	0.48 (0.43–0.53)	0.61 (0.55–0.68)	0.60 (0.49–0.73)	0.46 (0.38–0.56)	0.62 (0.51–0.77)	0.86 (0.66–1.13)	< 0.01	78%
*Age at first delivery*								
≤ 20 years	0.96 (0.85–1.09)	0.79 (0.69–0.90)	0.89 (0.69–1.14)	0.76 (0.62–0.92)	0.75 (0.57–0.97)	0.73 (0.46–1.17)	0.73	0%
21–25 years	Reference	Reference	Reference	Reference	Reference	Reference		
26–30 years	1.25 (1.16–1.33)	1.24 (1.15–1.33)	1.29 (1.05–1.58)	1.44 (1.26–1.64)	1.28 (1.13–1.45)	1.07 (0.92–1.23)	0.02	68%
> 30 years	1.61 (1.44–1.79)	1.52 (1.37–1.70)	1.33 (0.93–1.90)	1.62 (1.28–2.04)	1.77 (1.45–2.15)	1.31 (1.08–1.60)	0.15	43%
Nulliparous	2.35 (2.11–2.61)	1.76 (1.58–1.97)	1.74 (1.40–2.16)	2.40 (1.96–2.93)	1.77 (1.43–2.20)	1.23 (0.92–1.63)	< 0.01	80%
*Smoking*								
Never	Reference	Reference	Reference	Reference	Reference	Reference		
Ever	1.17 (1.05–1.31)	1.13 (1.01–1.26)	1.25 (0.99–1.58)	1.06 (0.85–1.32)	0.86 (0.69–1.08)	1.45 (1.14–1.84)	0.01	72%
*Alcohol consumption*								
Non-drinker	Reference	Reference	Reference	Reference	Reference	Reference		
Current drinker	1.03 (0.95–1.12)	1.15 (1.05–1.26)	1.10 (0.90–1.33)	1.15 (0.96–1.38)	1.07 (0.91–1.26)	1.22 (1.01–1.48)	0.77	0%

^a^The menopause age factor was only analysed among post-menopausal women

HR^1^: Hazard ratio adjusted for baseline age and age at first delivery

HR^2^: Hazard ratio adjusted for baseline age, age at first delivery, and cohort

A *P* value of the Q test < 0.1 or I^2^ > 50% implied a substantial difference by the stratified factors

The results of the meta-analysis of the association between each variable and breast cancer risk showed no notable heterogeneity except for the analysis of parity (*P* value of Q test = 0.02 and I2 = 52%). However, the random effects model result (0.58 [0.50–0.67]) was comparable to the pooled analysis result (Additional file 1: Figures S1–S5).

### Breast cancer risk according to birth cohorts

The results of the stratified analysis by birth cohort showed associations between reproductive factors and breast cancer risk among participants who were born in the 1920s or earlier, 1930s (except for menopause age) and 1940s, with the same directions as the overall analysis. However, no association was identified between smoking and alcohol consumption and breast cancer risk among these birth cohorts. Among the participants born in the 1950s or later, associations between smoking (1.45 [1.14–1.84]) and alcohol consumption (1.22 [1.01–1.48]) and a higher risk of breast cancer were observed. Furthermore, in this younger birth cohort, a menarche age < 13 years (1.30 [1.01–1.68]) and age at first delivery > 30 years (1.31 [1.08–1.60]) were also observed to increase the risk of breast cancer.

For some factors, the observed effect sizes for breast cancer risk varied across the birth cohorts. For a younger menarche age, the HRs for breast cancer varied between 1.24 and 1.55 (< 13 years), and between 1.12 and 1.47 (13–14 years). HRs for breast cancer appeared to be between 0.65 and 0.98 for a menopause age < 45 years, 0.75 and 1.24 for a menopause age of 45–49 years, and 0.79 and 2.66 for a menopause age ≥ 55 years. Furthermore, the HRs for breast cancer among parous women fell between 0.46 and 0.86. Regarding older age at first delivery, the HRs varied between 1.07 and 1.44 for those aged 26–30 years and 1.31 and 1.77 for ages > 30 years. Among ever smokers, the HRs were between 0.89 and 1.46.

The results of the meta-analysis of the association between each variable and breast cancer risk in the youngest and oldest birth cohort groups showed no notable heterogeneity. Furthermore, the result of the fixed effect model appeared to be comparable to the main results. (Additional file 1: Figures S6–S10).

### Additional analyses

The results of the stratified analysis by menopausal status are presented in Additional file 1: Table S4. Associations between reproductive factors and breast cancer risk among both pre- and post-menopausal women were similar to the overall analysis. Furthermore, regarding lifestyle factors, the results had the same direction as the overall analysis among both pre- and post-menopausal women.

The results of the stratified analysis by country indicated substantial differences in breast cancer risk across the country (Additional file 1: Table S5). Therefore, we further performed an additional analysis of the association between reproductive factors, lifestyle factors, and breast cancer risk by including the country as the adjusting variable (Additional file 1: Table S6). In general, the findings of this model were comparable to the results of the main analysis in terms of significance and direction, except for the group with a younger age at first delivery in the 1920s cohort, which appeared disparately.

## Discussion

In this study, we aimed to determine whether there is a differential pattern of reproductive and lifestyle factors for breast cancer risk according to birth cohorts. The study findings showed associations between reproductive factors and breast cancer risk in all birth cohorts; however, the associations of lifestyle factors were only notable in the later birth cohorts. The risk of breast cancer was observed to differ substantially across birth cohorts among parous women, those who were 26–30 years old at first delivery and those who had ever smoked.

In the overall pooled analysis, we observed associations between all reproductive factors and breast cancer risk, which was an expected result and is consistent with results from previous studies [30–33]. However, evidence of lifestyle factors, such as smoking and alcohol consumption, and their associations with breast cancer risk were inconsistent in Asia. Results from previous studies, which used data from the cohort studies that were included in this study, showed no association between active smoking and breast cancer risk [34, 35], similar to a meta-analysis among the Chinese [36]. However, other, more recent meta-analyses that involved more articles [37, 38] and a study with a larger sample size [39] reported contradictory findings. The contribution of alcohol drinking and the dose–response association to an elevated risk of breast cancer has been widely suggested [40–43]. However, this association was not apparent in the present study, which included only Asians [44–46]. A meta-analysis by Sun et al. [43] found an association between alcohol consumption and breast cancer in the overall analysis; however, it was not notable among the Asian population in their subgroup analysis, and articles from Asia had the lowest attributable percentages compared to those of North America and Europe. In this study, we confirmed positive associations between ever smoking and current drinking and an increased risk of breast cancer.

Many previous studies suggested that the birth cohort effect shifted the trends of reproductive and lifestyle factors, which subsequently contributed to the increasing rate of breast cancer in each corresponding country [5, 7, 30]. A previous study even suggested differences in breast cancer risk according to birth year [30]. In this study, we added evidence of a differential pattern of breast cancer risk, which was notable for some of the factors. Parous women were observed to have a lower breast cancer risk in the older generations and not the younger generation (1950s-born participants), with a higher magnitude observed among the 1930s birth cohort. Furthermore, this study’s analyses of lifestyle factors resulted in disparate associations across the birth cohorts. The associations between smoking and breast cancer only appeared in the younger generation group and not the older generation groups. Similarly, alcohol consumption appeared to have the same pattern as smoking.

The differing risk of breast cancer that appeared in the younger birth cohort in our study might be caused by the changes in prevalence of some factors which corresponds to the report by previous studies. A previous study reported a decreased number of parity [12], which may explain our finding that the association between parity and breast cancer risk was attenuated in the younger birth cohort. Furthermore, the increased number of women who smoke may explain the prominent association, with a higher effect size, between smoking and breast cancer risk in the younger birth cohort in the present study. Moreover, the difference between the ≥ 1950s birth cohort and the other older birth cohorts is supported by a previous study that concluded the presence of birth cohort effects based on the timing of westernisation in several Asian countries [47]. According to the study, Japan and Korea were assumed to have begun westernisation in the mid-1940s, whereas the timing in China varied according to the region [47]. This may explain the difference in breast cancer risk between participants born earlier (the 1920s to 1940s) and later.

Statistical significance is dependent on the sample size, and thus, significance might be affected by a large study population [48]. Therefore, interpreting the results using effect sizes rather than solely assessing significance may be justified because effect size is independent of sample size [48]. In our study, apart from the different associations found across birth cohorts, the effect sizes of several factors on the risk of breast cancer tended to differ according to birth cohort. Compared to the older birth cohorts, in the ≥ 1950s birth cohort, the effect size was lower for parous women (HR = 0.86 compared to 0.60, 0.46 and 0.62 in the ≤ 1920s, 1930s and 1940s respectively), higher for smoking (HR = 1.45 compared to 1.25, 1.06 and 0.89 in the ≤ 1920s, 1930s and 1940s respectively), and slightly higher for alcohol consumption (HR = 1.22 compared to 1.10, 1.15 and 1.07 in the ≤ 1920s, 1930s and 1940s respectively).

This study had some limitations. First, the information was collected by self-report questionnaires for most variables. Second, the number of participants who were ex-drinkers was small, and some cohorts did not provide this information; thus, we could not perform an analysis of past drinking habits and had to merge the never drinkers and past drinkers into the same category. Third, detailed information on breast cancer type was unavailable, which limited the analysis to the overall breast cancer cases only, whereas the risk pattern might also differ according to the breast cancer type. Fourth, we did not include other factors that could possibly influence the risk of breast cancer, such as different dietary patterns in each country and cohort study; therefore, further study is suggested to examine their influence. Fifth, we were unable to include all cohort studies from the Asia Cohort Consortium in this study. Only 12 cohort studies from three countries, which provided information on the variable of interest and agreed to participate, were included. This suggests that expanded research on Asians is needed. Finally, in the pooled data, only a few studies provided information regarding the number of cigarettes and the amount of alcohol consumed by the participants. Therefore, we could not perform a dose–response analysis in this study. Further study is recommended to assess the differential pattern of breast cancer risk factors by including the breast cancer subtype. Nevertheless, this study has the strength of the use of a large-scale pooled analysis of prospective studies in several Asian countries to study the differential pattern of reproductive and lifestyle risk factors of breast cancer. Moreover, to the best of our knowledge, the present study is by far the largest to assess the association between smoking and breast cancer risk among Asian women.

## Conclusions

In conclusion, in this study of a large sample of Asian women, positive associations between reproductive factors and breast cancer risk were observed, consistent with previous reports. We also added evidence regarding the associations of smoking and alcohol consumption with the increased risk of breast cancer among Asian women. Furthermore, we observed differential patterns of parity, smoking and alcohol consumption across birth cohorts. Reproductive risk factors were more apparent in the older birth cohorts, whereas smoking and alcohol use were only notable in the younger generation.

### Supplementary Information

**Additional file 1**. Supplementary material.

## Data Availability

The data that support the findings of this study were obtained from the Asia Cohort Consortium upon request. The data are not publicly available to protect the privacy of the individuals who participated in the study and can be accessed on request from the National Cancer Center Japan.
