# Supplementary material for: Differential patterns of reproductive and lifestyle risk factors for breast cancer according to birth cohorts among women in China, Japan and Korea
Source: Breast Cancer Res. 2024 Jan 22;26:15. doi: 10.1186/s13058-024-01766-0 (PMC10801993; doi:10.1186/s13058-024-01766-0)
Supplement: Supplementary file 1 — Additional file 1. Supplementary material. [file 13058_2024_1766_MOESM1_ESM.docx]

Supplementary Table S1. Participant selection

|  | Before exclusion | | | Exclusion 0 | | | Exclusion 1 | | Exclusion 2 | | Exclusion 3 | | | | | |
| --- | --- | --- | --- | --- | --- | --- | --- | --- | --- | --- | --- | --- | --- | --- | --- | --- |
| Cohort | N | Case | Men | | Missing gender | N remaining | Missing age | N remaining | Missing information on pregnancy status and/or number of delivery/parity | N remaining | Missing information on follow up duration* | Follow up duration <= 0 days | Follow up duration 0+ <6 month | N remaining | Case |  |
|  |  |  |  | |  |  |  |  |  |  |  |  |  |  |  |  |
| SWHS | 74,940 | 1,596 |  | |  | 74,940 |  | 74,940 | - | 74,940 | 3 |  | 103 | 74,937 | 1,596 |  |
| JPHC1 | 43,096 | 494 | 20,627 | |  | 22,469 |  | 22,469 | 965 | 21,504 | 29 | 3 | 41 | 21,472 | 467 |  |
| JPHC2 | 56,572 | 474 | 26,817 | |  | 29,755 |  | 29,755 | 1,992 | 27,763 | 28 | 1 | 38 | 27,734 | 450 |  |
| JACC | 86,505 | 280 | 36,199 | |  | 50,306 |  | 50,306 | 4,647 | 45,659 | 10 |  | 140 | 45,649 | 259 |  |
| LSS | 52,883 | 619 | 20,390 | |  | 32,493 | 2,416 | 30,077 | - | 30,077 |  | 27 | 42 | 29,380 | 476 |  |
| Miyagi | 47,605 | 561 | 22,836 | |  | 24,769 |  | 24,769 | 1,931 | 22,838 | 32 | 1 | 37 | 22,805 | 509 |  |
| Ohsaki | 51,253 | 247 | 24,573 | | 1 | 26,679 |  | 26,679 | 4,488 | 22,191 | 16 | 1 | 607 | 22,174 | 204 |  |
| KNCC | 42,751 | 259 | 21,425 | |  | 21,326 |  | 21,326 | 3,118 | 18,208 | 1,322 |  | 24 | 16,886 | 230 |  |
| Takayama | 31,552 | 202 | 14,427 | |  | 17,125 |  | 17,125 | 415 | 16,710 | 17 |  | 83 | 16,693 | 183 |  |
| 3pref-Miyagi | 31,345 | 123 | 13,992 | |  | 17,353 |  | 17,353 | 829 | 16,524 | 4 |  | 115 | 16,520 | 120 |  |
| 3pref-Aichi | 33,529 | 179 | 15,746 | |  | 17,783 |  | 17,783 | 17,783 | - |  |  |  |  |  |  |
| KMCC | 20,636 | 96 | 8,232 | | 5 | 12,399 |  | 12,399 | 961 | 11,438 | 1 | 19 | 25 | 11,418 | 63 |  |
| Namwon | 10,667 | 25 | 4,201 | |  | 6,466 |  | 6,466 | 176 | 6,290 | 3 |  | 6 | 6,287 | 24 |  |
|  |  |  |  | |  |  |  |  |  | - |  |  |  |  |  |  |
| Total | 583,334 | 5,155 | 229,465 | | 6 | 353,863 | 2,416 | 351,447 | 37,305 | 314,142 | 1,465 | 52 | 1,261 | 311,955 | **4,581** |  |

Supplementary Table S2. Comparison of included and excluded participants

|  | Total  N (%) | Included  N (%) | Excluded  N (%) | Standardized difference |
| --- | --- | --- | --- | --- |
| Age at baseline (years, mean) | 54.4 ± 10.9 | 54.0 ± 10.7 | 57.5 ± 11.2 | 0.317 |
| Menopausal status |  |  |  |  |
| Premenopausal | 10,9275 (31.1) | 103,195 (33.1) | 6,080 (15.4) | 0.357 |
| Postmenopausal | 220,169 (62.6) | 194,390 (62.3) | 25,779 (65.3) |  |
| Unknown | 22,003 (6.3) | 14,370 (4.6) | 7,633 (19.3) |  |
| Age at menarche |  |  |  | 0.118 |
| <13 years | 20,421 (5.8) | 19,766 (6.3) | 655 (1.7) |  |
| 13-14 years | 105,874 (30.1) | 102,516 (32.9) | 3,358 (8.5) |  |
| 15-16 years | 111,102 (31.6) | 107,179 (34.4) | 3,923 (9.9) |  |
| 17+ years | 58,172 (16.6) | 55,673 (17.8) | 2,499 (6.3) |  |
| Missing | 55,878 (15.9) | 26,821 (8.6) | 29,057 (73.6) |  |
| Age at menopause^a^ |  |  |  | 0.067 |
| <45 years | 29,386 (13.3) | 28,040 (14.4) | 1,346 (5.2) |  |
| 45-49 years | 64,403 (29.3) | 61,684 (31.7) | 2,719 (10.5) |  |
| 50-54 years | 78,303 (35.6) | 74,612 (38.4) | 3,691 (14.3) |  |
| 55+ years | 9,208 (4.2) | 8,711 (4.5) | 497 (1.9) |  |
| Missing | 38,869 (17.7) | 21,343 (11.0) | 17,526 (68.0) |  |
| Parity |  |  |  | 0.297 |
| Nulliparous | 21,442 (6.8) | 21,090 (6.8) | 352 (16.1) |  |
| Parous | 292,700 (93.2) | 290,865 (93.2) | 1,835 (83.9) |  |
| Number of children |  |  |  | 0.643 |
| 0 | 12,632 (3.6) | 12,601 (4.0) | 31 (0.1) |  |
| 1-2 | 149,716 (42.6) | 148,522 (47.6) | 1194 (3.0) |  |
| 3-4 | 94,204 (26.8) | 93,958 (30.1) | 246 (0.6) |  |
| 5+ | 26,618 (7.6) | 26,601 (8.5) | 17 (0.0) |  |
| Missing | 68,277 (19.4) | 30,273 (9.7) | 38004 (96.2) |  |
| Age at first delivery (%) |  |  |  | 0.457 |
| ≤20 years | 29,106 (9.5) | 28,979 (9.5) | 127 (0.3) |  |
| 21-25 years | 146,661 (47.8) | 14,6015 (47.9) | 646 (1.6) |  |
| 26-30 years | 90,925 (29.7) | 90,182 (29.6) | 743 (1.9) |  |
| >30 years | 18,472 (6.0) | 18,275 (6.0) | 197 (0.5) |  |
| Nulliparous | 21,442 (7.0) | 21,090 (6.9) | 352 (0.9) |  |
| Missing | 66,406 (17.8) | 28,979 (8.7) | 37,427 (94.8) |  |
| Breastfeeding among parous |  |  |  | 0.081 |
| Yes | 104,337 (35.65) | 103,181 (35.47) | 1,156 (63.00) |  |
| No | 16,051 (5.48) | 15,828 (5.44) | 223 (12.15) |  |
| Missing | 172,312 (58.87) | 171,856 (59.08) | 456 (24.85) |  |
| Smoking status (%) |  |  |  | 0.218 |
| Never | 291,564 (82.96) | 264,230 (84.70) | 27,334 (69.21) |  |
| Ever | 27,857 (7.93) | 23,058 (7.39) | 4,799 (12.15) |  |
| Missing | 32,026 (9.11) | 2,4667 (7.91) | 7,359 (18.63) |  |
| Alcohol drinking (%) |  |  |  | 0.336 |
| Non-drinker^b^ | 228,576 (65.0) | 208,535 (66.8) | 20,041 (50.7) |  |
| Current drinker | 64,638 (18.4) | 53,682 (17.2) | 10,956 (27.7) |  |
| Missing | 58,233 (16.6) | 49,738 (15.9) | 8,495 (21.5) |  |

Standardised differences were calculated using Cohen’s effect size classifications for small, medium, large and very large effect sizes (0.2, 0.5, 0.8 and 1.3, respectively) without including the number of missing information. Absolute values of standardised differences >0.5 were considered large imbalances.

^a^The result regarding menopause age was based on the analysis of post-menopausal women.

^b^The non-drinker category of alcohol consumption included both participants who never drank alcohol and ex-drinkers.

Supplementary Table S3. Distributions of baseline characteristics in each cohort

|  | Total | SWHS | JPHC 1 | JPHC2 | JACC | LSS | Miyagi | Ohsaki | KNCC | Takayama | 3 Pref Miyagi | KMCC | Namwon |
| --- | --- | --- | --- | --- | --- | --- | --- | --- | --- | --- | --- | --- | --- |
| Women at baseline (N) | 311 955 | 74 937 | 21 472 | 27 734 | 45 649 | 29 380 | 22 805 | 22 174 | 16 886 | 16 693 | 16 520 | 11 418 | 6 287 |
| Breast cancer cases (N) | 4,581 | 1,596 | 467 | 450 | 259 | 476 | 509 | 204 | 230 | 183 | 120 | 63 | 24 |
| Baseline year (min-max) |  | 1996-2000 | 1990-1992 | 1993-1995 | 1988-1990 | 1963-1992 | 1990-1990 | 1996-1996 | 2002-2015 | 1992-1992 | 1984  -1984 | 1993-2005 | 2004-2007 |
| Follow-up duration (years, mean) | 16.5 | 17.2 | 21.3 | 18.3 | 16.3 | 22.3 | 22 | 10.9 | 9.1 | 13.9 | 7.7 | 14.6 | 12.7 |
| SD | 6.6 | 3.3 | 4.0 | 3.6 | 5.6 | 10.4 | 5.6 | 4.3 | 3.3 | 3.9 | 2.5 | 4.4 | 2.1 |
| Follow-up duration among breast cancer cases (years, mean) | 10.2 | 9.6 | 11.4 | 10.6 | 7.2 | 16.4 | 12.4 | 6.8 | 5.3 | 8.5 | 4.1 | 7.9 | 6.3 |
| SD | 6.6 | 5.1 | 6.5 | 5.8 | 5.1 | 9.2 | 6.9 | 3.9 | 3.6 | 4.4 | 2.6 | 4.6 | 3.4 |
| Age at baseline (years, mean) | 54.2 | 52.6 | 49.6 | 54.3 | 57.4 | 51.8 | 52.2 | 60.5 | 49.8 | 56.3 | 57.4 | 54 | 61.2 |
| SD | 10.7 | 9.1 | 5.9 | 8.8 | 9.9 | 15.0 | 7.4 | 10.0 | 9.0 | 13.2 | 11.3 | 14.2 | 7.9 |
| Age at baseline among breast cancer cases (years, mean) | 51.7 | 52.0 | 49.4 | 52.4 | 55.8 | 48.2 | 50.9 | 57.9 | 49 | 52.8 | 56.7 | 49.3 | 55.7 |
| SD | 9.3 | 8.7 | 5.8 | 8.6 | 9.5 | 12.4 | 7.3 | 10.1 | 7.9 | 11.3 | 9.7 | 10.3 | 7.2 |
| Age at diagnosis of breast cancer cases (years, mean) | 61.9 | 61.6 | 60.8 | 63.0 | 63.0 | 64.6 | 63.3 | 64.6 | 54.3 | 61.3 | 60.8 | 57.2 | 62.0 |
| SD | 10.4 | 9.8 | 8.8 | 10.0 | 10.8 | 12.5 | 10.1 | 10.8 | 8.1 | 12.2 | 9.9 | 11.8 | 7.4 |
| Birth year (%) |  |  |  |  |  |  |  |  |  |  |  |  |  |
| ≤1920s | 23.0 | 2.5 | 0 | 18.6 | 43.2 | 64.7 | 17.1 | 29.4 | 0.1 | 31.1 | 54.6 | 11.7 | 0 |
| 1930s | 29.7 | 26.5 | 47.1 | 34.0. | 32.2 | 20.4 | 41.0 | 38.5 | 1.5 | 23.2 | 32.0 | 28.6 | 31.2 |
| 1940s | 25.9 | 25.4 | 46.7 | 31.0 | 24.2 | 14.9 | 37.0 | 19.1 | 16.6 | 27.3 | 13.4 | 25.5 | 40.1 |
| ≥1950s | 21.4 | 45.6 | 6.2 | 16.4 | 0.5 | 0 | 4.9 | 13 | 81.8 | 18.4 | 0 | 34.1 | 28.8 |
| Premenopausal women (%) | 33.1 | 49.5 | 44.7 | 32.4 | 11.5 | 40.4 | 38.5 | 17.3 | 35.4 | 37.9 | 26.7 | 2.5 | 12.0 |
| unknown (%) | 4.6 | 0 | 0.2 | 0.2 | 14.6 | 7.0 | 1.0 | 0.4 | 12.4 | 1.2 | 2.2 | 22.6 | 0 |
| Age at menarche (%) |  |  |  |  |  |  |  |  |  |  |  |  |  |
| <13 years | 6.3 | 6.2 | 8.8 | 9.0 | 6.3 | 4.4 | 7.8 | 2.2 | 7.0 | 12.0 | 5.4 | 1.2 | 1.3 |
| 13-14 years | 32.9 | 36.5 | 40.8 | 39.0 | 35.0 | 29.0 | 37.0 | 15.1 | 34.8 | 35.2 | 32.4 | 12.6 | 12.3 |
| 15-16 years | 34.4 | 39.4 | 34.9 | 32.6 | 36.5 | 27.5 | 31.2 | 22.6 | 36.1 | 35.2 | 38.4 | 32.4 | 34.5 |
| 17+ years | 17.8 | 17.8 | 14.1 | 18.3 | 18.0 | 12.1 | 13.2 | 11.5 | 15.8 | 14.8 | 17.8 | 49.7 | 50.8 |
| Missing | 8.6 | 0.1 | 1.3 | 1.1 | 4.2 | 27.0 | 10.8 | 48.7 | 6.2 | 2.8 | 6.1 | 4.0 | 1.2 |
| Age at menopause^a^ (%) |  |  |  |  |  |  |  |  |  |  |  |  |  |
| <45 years | 16.2 | 16.8 | 16.1 | 14.6 | 13.6 | 17.8 | 18.8 | 14.2 | 15.1 | 14.2 | 16.1 | 23.9 | 25.1 |
| 45-49 years | 35.6 | 43.9 | 37.2 | 32.9 | 33.1 | 37.8 | 33.8 | 31.9 | 30.5 | 28.8 | 365 | 30.5 | 32.6 |
| 50-54 years | 43.1 | 35.4 | 44.0 | 47.4 | 48.0 | 40.3 | 43.2 | 47.6 | 45.6 | 51.7 | 43.0 | 37.2 | 35.0 |
| 55+ years | 5 | 3.9 | 2.6 | 5.2 | 5.3 | 4.1 | 4.2 | 6.3 | 8.7 | 5.3 | 4.4 | 8.4 | 7.3 |
| Parous (%) | 93.2 | 96.7 | 94.5 | 94.2 | 96.1 | 71.1 | 97.5 | 96.7 | 96.6 | 91.2 | 90.3 | 95.4 | 99.4 |
| Number of children (%) |  |  |  |  |  |  |  |  |  |  |  |  |  |
| 0 | 4.0 | 3.3 | 5.5 | 5.8 | 3.9 | 0 | 2.5 | 3.3 | 3.4 | 8.8 | 9.7 | 4.7 | 0.6 |
| 1-2 | 47.6 | 75.7 | 41.7 | 40.2 | 44.4 | 0 | 48.3 | 41.2 | 72.4 | 56.1 | 39.0 | 21.5 | 13.4 |
| 3-4 | 30.1 | 16.7 | 43.1 | 39.3 | 43.5 | 0 | 45.2 | 45.2 | 22.3 | 29.3 | 34.2 | 36.0 | 42.8 |
| 5+ | 8.5 | 4.3 | 8.8 | 13.6 | 8.2 | 0 | 4.0 | 10.3 | 1.9 | 5.8 | 17.1 | 34.7 | 43.2 |
| Missing | 9.7 | 0 | 0.9 | 1.2 | 0 | 100.0 | 0 | 0 | 0 | 0 | 0 | 3.2 | 0 |
| Age at first delivery (%) |  |  |  |  |  |  |  |  |  |  |  |  |  |
| ≤20 years | 9.3 | 11.4 | 7.8 | 6.8 | 4.9 | 15.9 | 7.2 | 8.3 | 2.1 | 4.9 | 9.3 | 19.0 | 25.6 |
| 21-25 years | 46.8 | 30.6 | 50.2 | 52.6 | 52.0 | 39.4 | 64.8 | 65.7 | 34.6 | 50.6 | 54.2 | 53.1 | 60.7 |
| 26-30 years | 28.9 | 44.3 | 28.4 | 26.1 | 28.5 | 12.5 | 21.5 | 16.5 | 48.4 | 27.3 | 19.8 | 15.1 | 10.9 |
| >30 years | 5.9 | 10.4 | 5.9 | 4.9 | 4.4 | 3.3 | 3.4 | 2.7 | 8.5 | 5.3 | 4.7 | 2.4 | 1.7 |
| Nulliparous | 6.8 | 3.3 | 5.5 | 5.8 | 3.9 | 28.9 | 2.5 | 3.3 | 3.4 | 8.8 | 9.7 | 4.7 | 0.6 |
| Breastfeeding among parous women (%) | 35.5 | 0 | 84.3 | 86.3 | 0 | 0 | 78.9 | 84.0 | 76.6 | 0 | 0 | 86.8 | 97.0 |
| Missing | 59.1 | 10 | 1.7 | 2.6 | 100 | 100 | 3.1 | 1.9 | 8.5 | 100 | 100 | 9.3 | 0.1 |
| Smoking status (%) |  |  |  |  |  |  |  |  |  |  |  |  |  |
| Never | 84.7 | 97.2 | 92.2 | 91.8 | 83.4 | 79.6 | 67.2 | 72.1 | 86.3 | 74.2 | 61.1 | 90.8 | 94.7 |
| Ever | 7.4 | 2.8 | 7.4 | 7.7 | 5.7 | 14.6 | 8.2 | 8.3 | 7.4 | 15.7 | 8.7 | 8.4 | 5.3 |
| Missing | 7.9 | 0 | 0.3 | 0.6 | 10.9 | 5.8 | 24.6 | 19.6 | 6.3 | 10.1 | 30.2 | 0.7 | 0 |
| Alcohol drinking (%) |  |  |  |  |  |  |  |  |  |  |  |  |  |
| Non-drinker^b^ | 66.8 | 98.1 | 77.0 | 78.9 | 71.3 | 73.8 | 60.6 | 64.7 | 55.7 | - | 48.6 | 80.9 | 65.3 |
| Current drinker | 17.2 | 1.9 | 23.0 | 19.8 | 21.9 | 26.2 | 21.0 | 19.0 | 41.5 | - | 23.4 | 18.2 | 34.2 |
| Missing | 15.9 | 0 | 0 | 1.2 | 6.9 | 0 | 18.4 | 16.3 | 2.8 | 100.0 | 28.0 | 0.9 | 0.4 |

^a^The result regarding menopause age was based on the analysis of post-menopausal women.

^b^The non-drinker category of alcohol consumption included both participants who never drank alcohol and ex-drinkers.

Supplementary Table S4. Associations of reproductive factors, smoking status and alcohol consumption with breast cancer risk based on menopausal status

|  | Overall | |  | Birth Year | | | |
| --- | --- | --- | --- | --- | --- | --- | --- |
|  | HR^1^ (95% CI) | HR^2^ (95% CI) |  | ≤1920s | 1930s | 1940s | ≥1950s |
|  |  |  |  | HR^2^ (95% CI) | HR^2^ (95% CI) | HR^2^ (95% CI) | HR^2^ (95% CI) |
| *Premenopausal women* |  |  |  |  |  |  |  |
| Menarche age |  |  |  |  |  |  |  |
| <13 years | 0.93 (0.76-1.15) | 1.22 (0.99-1.50) |  | 0.72 (0.28-1.82) | 1.48 (0.65-3.40) | 1.17 (0.80-1.72) | 1.39 (1.05-1.84) |
| 13-14 years | 1.01 (0.85-1.19) | 1.18 (0.99-1.40) |  | 0.67 (0.37-1.21) | 1.10 (0.62-1.97) | 1.36 (0.98-1.90) | 1.19 (0.94-1.50) |
| 15-16 years | 1.00 (0.84-1.20) | 1.08 (0.90-1.28) |  | 0.54 (0.29-1.02) | 1.02 (0.56-1.83) | 1.22 (0.87-1.71) | 1.13 (0.89-1.43) |
| 17+ years | Reference | Reference |  | Reference | Reference | Reference | Reference |
| (cont.) | 1.00 (0.97-1.03) | 0.96 (0.93-0.99) |  | 1.03 (0.91-1.17) | 0.94 (0.84-1.05) | 0.96 (0.91-1.01) | 0.96 (0.92-1.00) |
| Parity |  |  |  |  |  |  |  |
| Nulliparous | Reference | Reference |  | Reference | Reference | Reference | Reference |
| Parous | 0.54 (0.46-0.64) | 0.67 (0.57-0.79) |  | 0.87 (0.50-1.50) | 0.42 (0.28-0.65) | 0.62 (0.47-0.82) | 0.77 (0.58-1.02) |
| Age at first delivery |  |  |  |  |  |  |  |
| ≤20 years | 1.03 (0.81-1.32) | 0.84 (0.66-1.08) |  | 1.05 (0.57-1.93) | 1.17 (0.61-2.23) | 0.75 (0.51-1.1) | 0.8 (0.48-1.32) |
| 21-25 years | Reference | Reference |  | Reference | Reference | Reference | Reference |
| 26-30 years | 1.09 (0.98-1.20) | 1.15 (1.03-1.29) |  | 1.74 (1.02-2.97) | 1.19 (0.76-1.87) | 1.23 (1.04-1.46) | 1.04 (0.88-1.22) |
| >30 years | 1.38 (1.19-1.61) | 1.43 (1.22-1.68) |  | 0.68 (0.16-2.81) | 1.39 (0.60-3.23) | 1.61 (1.22-2.12) | 1.30 (1.05-1.61) |
| Nulliparous | 1.97 (1.66-2.35) | 1.61 (1.35-1.92) |  | 1.29 (0.72-2.32) | 2.56 (1.61-4.08) | 1.74 (1.31-2.31) | 1.35 (1.01-1.82) |
| Smoking |  |  |  |  |  |  |  |
| Never | Reference | Reference |  | Reference | Reference | Reference | Reference |
| Ever | 1.15 (0.97-1.35) | 1.16 (0.98-1.38) |  | 1.41 (0.82-2..42) | 1.11 (0.63-1.95) | 0.88 (0.66-1.16) | 1.47 (1.13-1.90) |
| Alcohol consumption |  |  |  |  |  |  |  |
| Non-current drinker | Reference | Reference |  | Reference | Reference | Reference | Reference |
| Current drinker | 1.04 (0.92-1.17) | 1.11 (0.97-1.28) |  | 0.91 (0.57-1.48) | 0.99 (0.65-1.50) | 1.08 (0.88-1.33) | 1.21 (0.97-1.52) |
| *Postmenopausal women* |  |  |  |  |  |  |  |
| Menarche age |  |  |  |  |  |  |  |
| <13 years | 1.61 (1.32-1.96) | 1.50 (1.23-1.83) |  | 1.69 (1.05-2.71) | 1.56 (1.16-2.10) | 1.41 (0.97-2.05) | 0.95 (0.43-2.10) |
| 13-14 years | 1.50 (1.33-1.69) | 1.40 (1.24-1.59) |  | 1.63 (1.26-2.09) | 1.29 (1.08-1.54) | 1.47 (1.12-1.93) | 0.89 (0.52-1.52) |
| 15-16 years | 1.26 (1.12-1.42) | 1.20 (1.06-1.35) |  | 1.16 (0.91-1.48) | 1.26 (1.07-1.49) | 1.14 (0.87-1.50) | 0.81 (0.48-1.37) |
| 17+ years | Reference | Reference |  | Reference | Reference | Reference | Reference |
| (cont.) | 0.92 (0.90-0.94) | 0.93 (0.91-0.95) |  | 0.89 (0.84-0.94) | 0.94 (0.91-0.98) | 0.94 (0.89-0.99) | 0.99 (0.89-1.10) |
| Parity |  |  |  |  |  |  |  |
| Nulliparous | Reference | Reference |  | Reference | Reference | Reference | Reference |
| Parous | 0.41 (0.36-0.47) | 0.55 (0.47-0.63) |  | 0.51 (0.41-0.64) | 0.46 (0.37-0.58) | 0.66 (0.46-0.95) | 0.97 (0.40-2.40) |
| Age at first delivery |  |  |  |  |  |  |  |
| ≤20 years | 0.94 (0.81-1.10) | 0.76 (0.65-0.89) |  | 0.87 (0.65-1.16) | 0.73 (0.59-0.9) | 0.75 (0.52-1.08) | 0.23 (0.03-1.65) |
| 21-25 years | Reference | Reference |  | Reference | Reference | Reference | Reference |
| 26-30 years | 1.34 (1.22-1.48) | 1.32 (1.20-1.46) |  | 1.20 (0.96-1.50) | 1.44 (1.25-1.66) | 1.34 (1.11-1.62) | 1.10 (0.73-1.65) |
| >30 years | 1.78 (1.51-2.09) | 1.61 (1.37-1.89) |  | 1.51 (1.04-2.18) | 1.64 (1.28-2.09) | 1.93 (1.44-2.58) | 1.01 (0.50-2.04) |
| Nulliparous | 2.73 (2.37-3.14) | 1.97 (1.70-2.29) |  | 2.01 (1.58-2.56) | 2.39 (1.90-3.00) | 1.67 (1.15-2.43) | 1.06 (0.41-2.70) |
| Smoking |  |  |  |  |  |  |  |
| Never | Reference | Reference |  | Reference | Reference | Reference | Reference |
| Ever | 1.15 (0.98-1.35) | 1.11 (0.95-1.30) |  | 1.20 (0.92-1.56) | 1.08 (0.85-1.38) | 0.92 (0.62-1.38) | 1.51 (0.75-3.04) |
| Alcohol consumption |  |  |  |  |  |  |  |
| Non-drinker | Reference | Reference |  | Reference | Reference | Reference | Reference |
| Current drinker | 0.95 (0.84-1.07) | 1.08 (0.95-1.23) |  | 1.11 (0.88-1.38) | 1.15 (0.93-1.42) | 0.96 (0.71-1.29) | 0.94 (0.59-1.49) |

HR^1^: Hazard ratio adjusted for baseline age and age at first delivery.

HR^2^: Hazard ratio adjusted for baseline age, age at first delivery, and cohort.

Supplementary Table S5. Associations of reproductive factors, smoking status and alcohol consumption with breast cancer risk according to country

|  |  | China  HR (95% CI) | Japan  HR (95% CI) | Korea  HR (95% CI) | Heterogeneity Test | |
| --- | --- | --- | --- | --- | --- | --- |
|  |  |  |  |  | P*-*value (Q test) | I^2^ |
| Menarche age |  |  |  |  |  |  |
| <13 years |  | 1.44 (1.15-1.81) | 1.03 (0.85-1.23) | 2.27 (1.39-3.73) | <0.01 | 83% |
| 13–14 years |  | 1.36 (1.16-1.59) | 1.15 (1.00-1.31) | 1.54 (1.10-2.15) | 0.12 | 52% |
| 15–16 years |  | 1.26 (1.08-1.48) | 1.06 (0.93-1.21) | 1.28 (0.94-1.74) | 0.21 | 37% |
| 17+ years |  | Reference | Reference | Reference |  |  |
| (cont.) |  | 0.94 (0.92-0.97) | 0.98 (0.96-1.01) | 0.89 (0.83-0.95) | 0.02 | 76% |
| Menopause age^a^ |  |  |  |  |  |  |
| <45 years |  | 0.79 (0.63-0.99) | 0.88 (0.75-1.04) | 0.71 (0.43-1.16) | 0.60 | 0% |
| 45–49 years |  | 0.80 (0.68-0.94) | 0.90 (0.80-1.02) | 0.82 (0.55-1.22) | 0.51 | 0% |
| 50–54 years |  | Reference | Reference | Reference |  |  |
| ≥55 years |  | 1.16 (0.82-1.65) | 0.99 (0.75-1.30) | 1.80 (1.07-3.11) | 0.38 | 0% |
| Parity |  |  |  |  |  |  |
| Nulliparous |  | Reference | Reference | Reference |  |  |
| Parous |  | 0.77 (0.60-0.99) | 0.37 (0.33-0.42) | 1.82 (0.98-3.39) | <0.01 | 96% |
| Age at first delivery |  |  |  |  |  |  |
| ≤20 years |  | 0.70 (0.57-0.87) | 1.09 (0.93-1.29) | 0.39 (0.21-0.70) | <0.01 | 89% |
| 21–25 years |  | Reference | Reference | Reference |  |  |
| 26–30 years |  | 1.20 (1.06-1.36) | 1.20 (1.09-1.32) | 1.75 (1.36-2.25) | 0.02 | 75% |
| >30 years |  | 1.54 (1.30-1.81) | 1.53 (1.30-1.81) | 1.73 (1.09-2.75) | 0.89 | 0% |
| Nulliparous |  | 1.45 (1.12-1.87) | 2.90 (2.57-3.27) | 0.84 (0.43-1.60) | <0.01 | 0% |
| Smoking |  |  |  |  |  |  |
| Never |  | Reference | Reference | Reference |  |  |
| Ever |  | 0.96 (0.69-1.34) | 1.15 (1.01-1.31) | 1.86 (1.30-2.67) | 0.02 | 74% |
| Alcohol consumption |  |  |  |  |  |  |
| Non-drinker |  | Reference | Reference | Reference |  |  |
| Current drinker |  | 0.90 (0.62-1.32) | 1.07 (0.97-1.18) | 1.40 (1.11-1.77) | 0.07 | 63% |

^a^The menopause age factor was only analysed among post-menopausal women.

HR: Hazard ratio adjusted for baseline age and age at first delivery.

A P-value of the Q test <0.1 or I^2^ >50% implied a substantial difference by the stratified factors.

Supplementary Table S6. Associations of reproductive factors, smoking status, and alcohol consumption with breast cancer risk, adjusted by country

|  | All  HR (95% CI) | Birth Cohorts | | | |  |  |
| --- | --- | --- | --- | --- | --- | --- | --- |
|  |  | ≤1920s  HR (95% CI) | 1930s  HR (95% CI) | 1940s  HR (95% CI) | ≥1950s  HR (95% CI) | Heterogeneity Test | |
|  |  |  |  |  |  | P*-*value (Q test) | I^2^ |
| Menarche age |  |  |  |  |  |  |  |
| <13 years | 1.31 (1.14-1.50) | 1.45 (0.96-2.19) | 1.38 (1.05-1.82) | 1.21 (0.93-1.57) | 1.45 (1.13-1.88) | 0.78 | 0% |
| 13–14 years | 1.32 (1.19-1.45) | 1.49 (1.19-1.87) | 1.16 (0.98-1.37) | 1.44 (1.17-1.77) | 1.24 (1.01-1.52) | 0.23 | 31% |
| 15–16 years | 1.17 (1.07-1.29) | 1.11 (0.89-1.38) | 1.15 (0.99-1.35) | 1.22 (0.99-1.51) | 1.14 (0.93-1.41) | 0.94 | 0% |
| 17+ years | Reference | Reference | Reference | Reference | Reference |  |  |
| (cont.) | 0.95 (0.93-0.97) | 0.91 (0.87-0.95) | 0.97 (0.93-1.00) | 0.95 (0.92-0.99) | 0.95 (0.91-0.98) | 0.13 | 47% |
| Menopause age^a^ |  |  |  |  |  |  |  |
| <45 years | 0.82 (0.72-0.93) | 0.72 (0.54-0.97) | 0.99 (0.82-1.21) | 0.85 (0.66-1.11) | 0.95 (0.46-1.97) | 0.37 | 5% |
| 45–49 years | 0.86 (0.78-0.94) | 0.83 (0.68-1.03) | 0.91 (0.79-1.05) | 0.79 (0.65-0.97) | 1.23 (0.69-2.22) | 0.44 | 0% |
| 50–54 years | Reference | Reference | Reference | Reference | Reference |  |  |
| ≥55 years | 1.12 (0.92-1.37) | 0.74 (0.49-1.11) | 1.24 (0.95-1.62) | 1.39 (0.85-2.29) | 2.79 (1.03-7.54) | 0.04 | 65% |
| Parity |  |  |  |  |  |  |  |
| Nulliparous | Reference | Reference | Reference | Reference | Reference |  |  |
| Parous | 0.47 (0.42-0.51) | 0.40 (0.33-0.49) | 0.38 (0.32-0.46) | 0.50 (0.41-0.61) | 0.86 (0.67-1.12) | <0.01 | 89% |
| Age at first delivery |  |  |  |  |  |  |  |
| ≤20 years | 0.94 (0.83-1.07) | 1.34 (1.04-1.73) | 0.81 (0.66-0.98) | 0.77 (0.59-1.00) | 0.66 (0.42-1.05) | <0.01 | 78% |
| 21–25 years | Reference | Reference | Reference | Reference | Reference |  |  |
| 26–30 years | 1.19 (1.11-1.27) | 1.12 (0.92-1.37) | 1.37 (1.20-1.56) | 1.25 (1.11-1.41) | 1.14 (0.98-1.32) | 0.21 | 34% |
| >30 years | 1.51 (1.36-1.68) | 1.35 (0.95-1.92) | 1.61 (1.28-2.04) | 1.69 (1.39-2.06) | 1.40 (1.15-1.70) | 0.49 | 0% |
| Nulliparous | 2.34 (2.11-2.61) | 2.67 (2.16-3.29) | 2.88 (2.36-3.49) | 2.19 (1.78-2.70) | 1.27 (0.96-1.70) | <0.01 | 87% |
| Smoking |  |  |  |  |  |  |  |
| Never | Reference | Reference | Reference | Reference | Reference |  |  |
| Ever | 1.22 (1.09-1.37) | 1.49 (1.18-1.89) | 1.12 (0.90-1.40) | 0.91 (0.72-1.14) | 1.55 (1.22-1.96) | <0.01 | 78% |
| Alcohol consumption |  |  |  |  |  |  |  |
| Non-drinker | Reference | Reference | Reference | Reference | Reference |  |  |
| Current drinker | 1.15 (1.05-1.25) | 1.14 (0.94-1.39) | 1.05 (0.88-1.26) | 1.07 (0.92-1.25) | 1.36 (1.13-1.63) | 0.17 | 41% |

^a^The menopause age factor was only analysed among post-menopausal women.

HR: Hazard ratio adjusted for baseline age and age at first delivery.

A P-value of the Q test <0.1 or I^2^ >50% implied a substantial difference by the stratified factors.


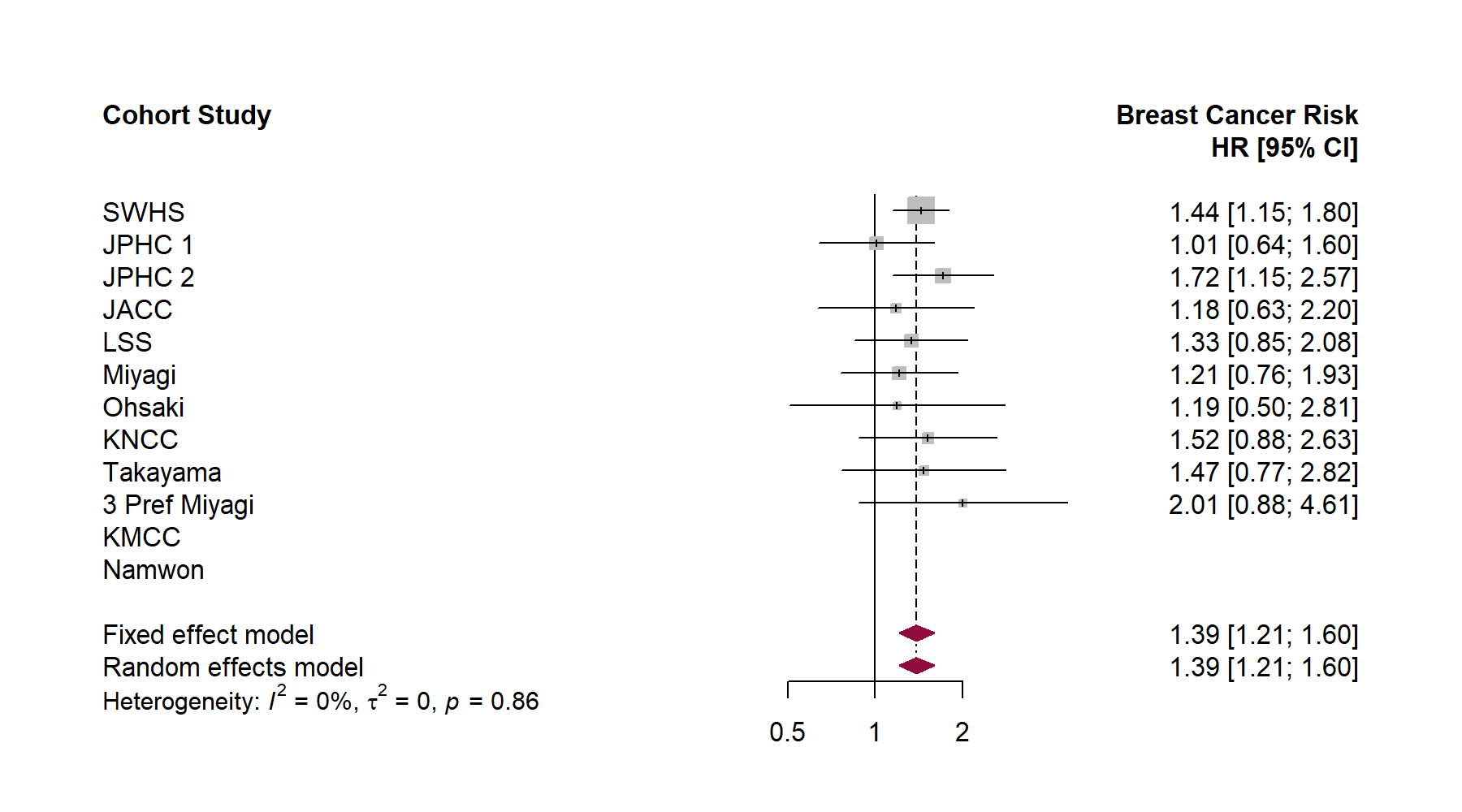


Supplementary Figure S1. Forest plot showing meta-analysis of hazard ratios for breast cancer risk in participants with menarche age <13 years (versus +17 years)


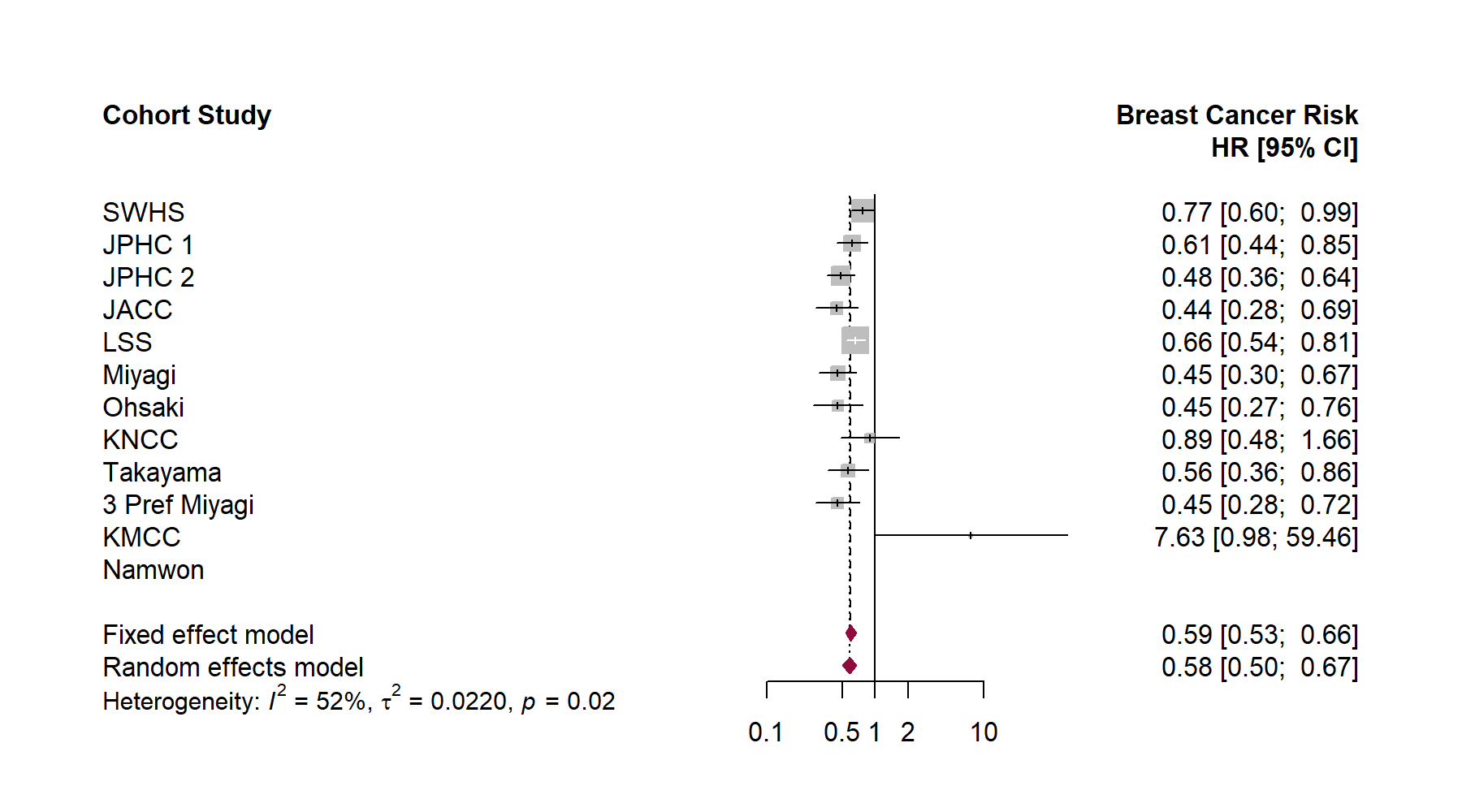


Supplementary Figure S2. Forest plot showing meta-analysis of hazard ratios for breast cancer risk in parous participants (versus nulliparous)


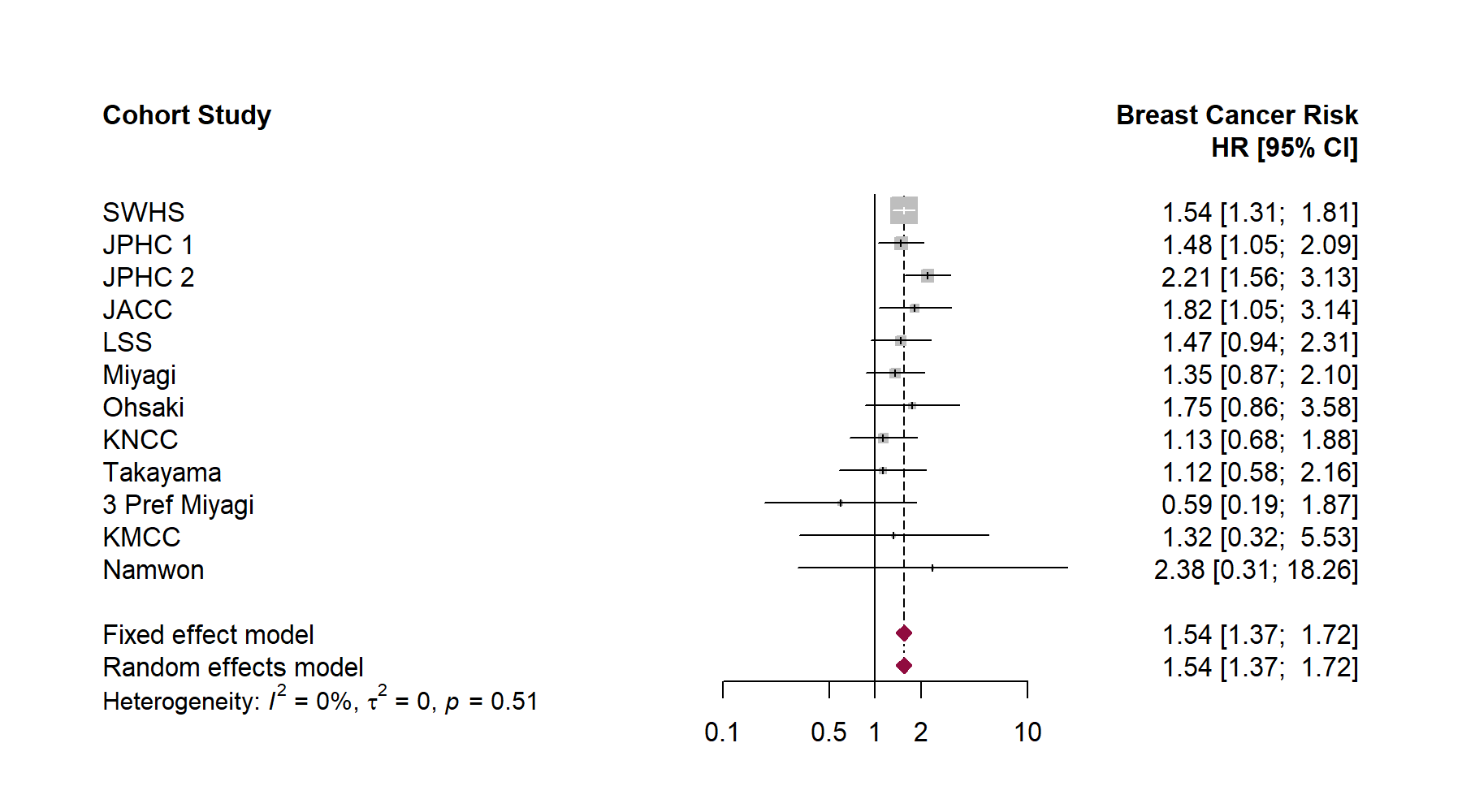


Supplementary Figure S3. Forest plot showing meta-analysis of hazard ratios for breast cancer risk in participants with an age at first delivery >30 years (versus 21-25 years)


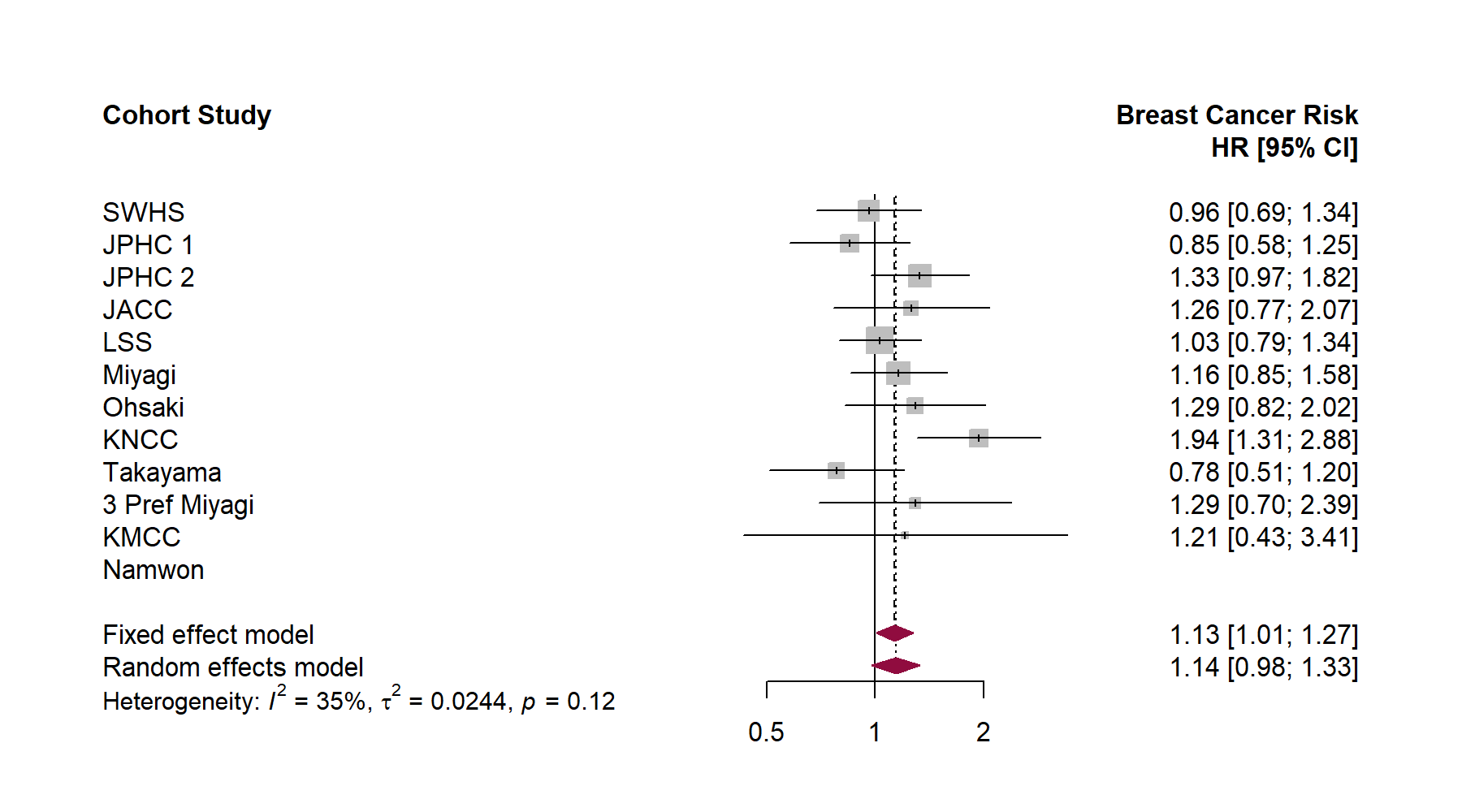


Supplementary Figure S4. Forest plot showing meta-analysis of hazard ratios for breast cancer risk in participants who ever smokers (versus never smokers)


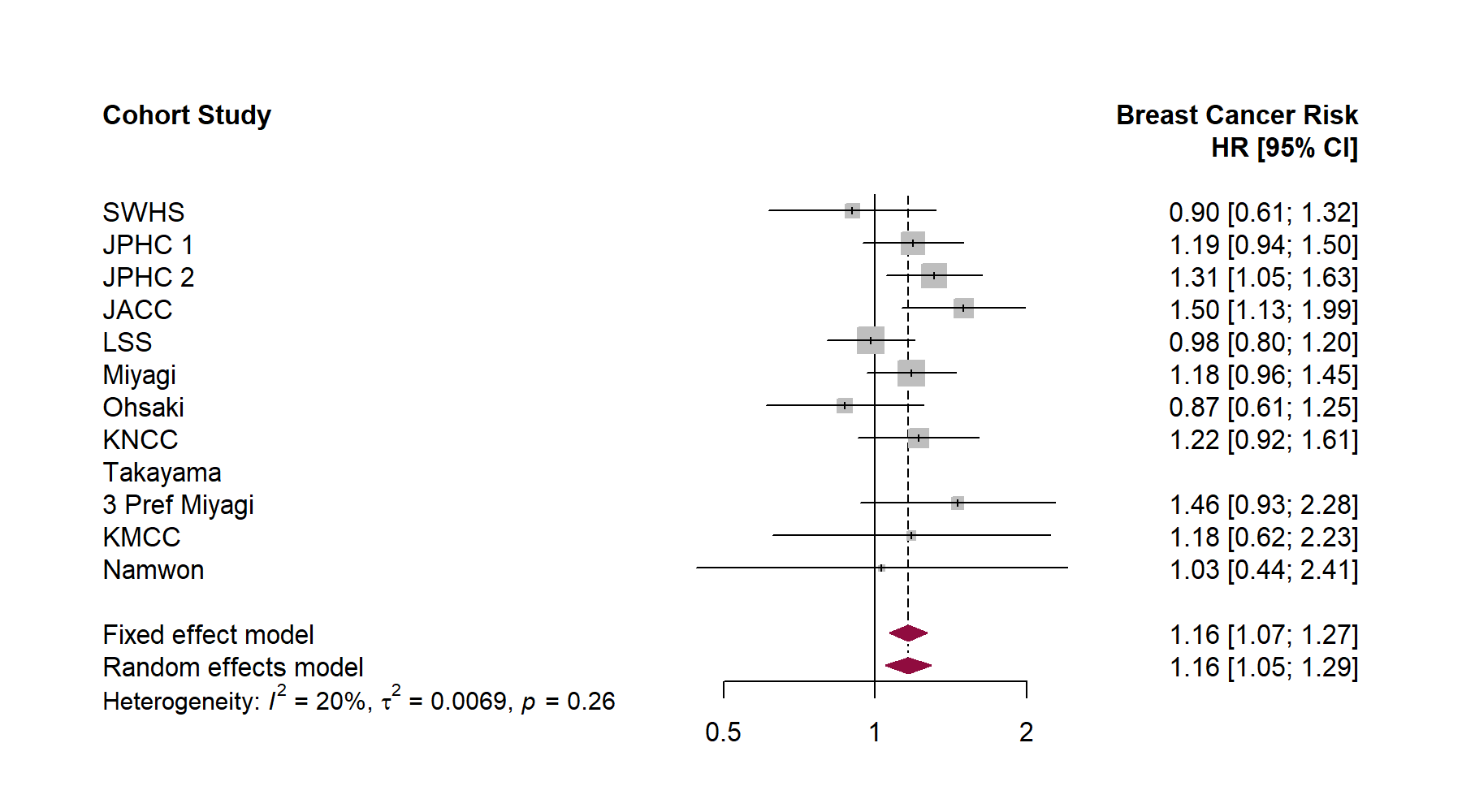


Supplementary Figure S5. Forest plot showing meta-analysis of hazard ratios for breast cancer risk in drinkers (versus non-drinkers)

| 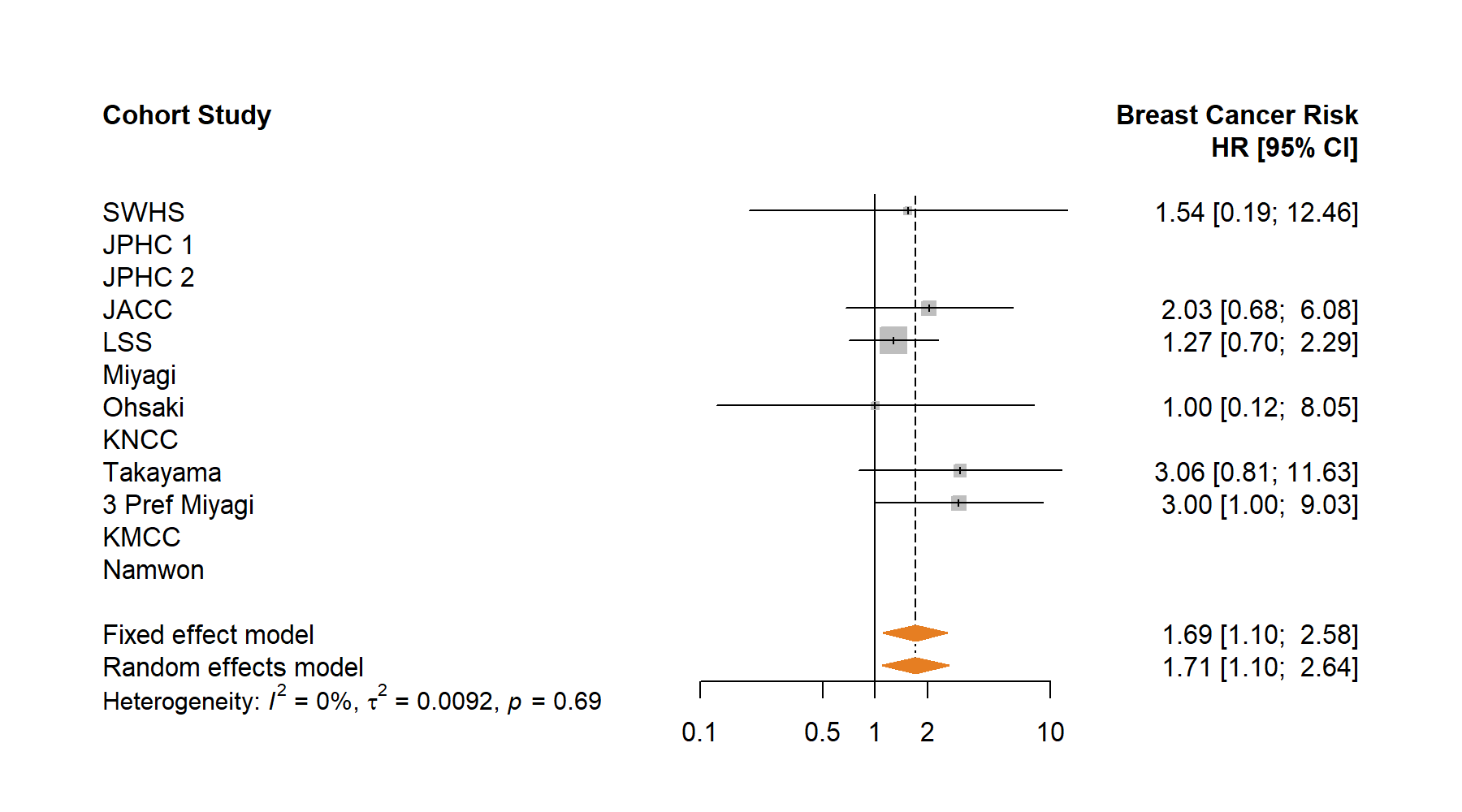  (b) | 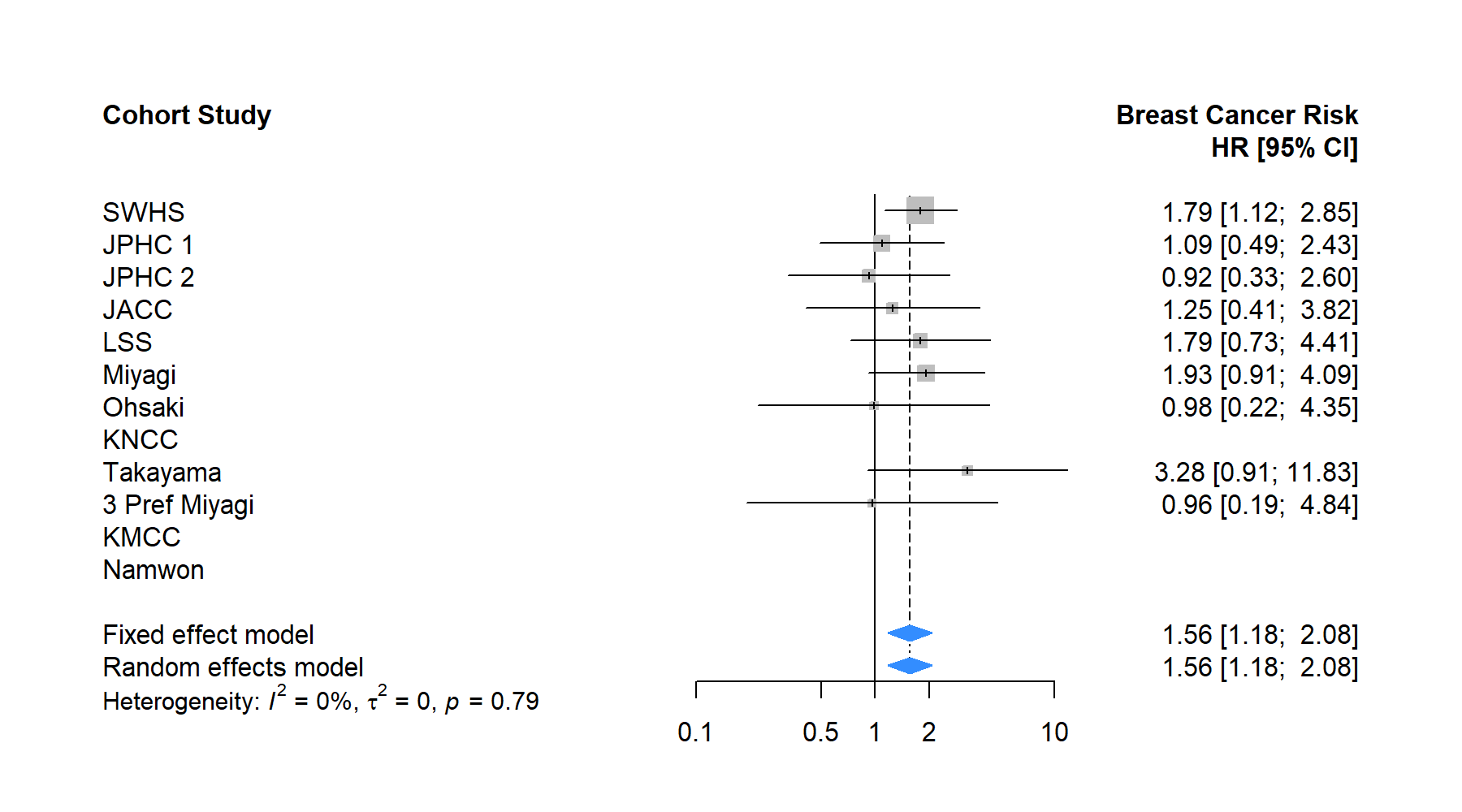  (c) |
| --- | --- |
| 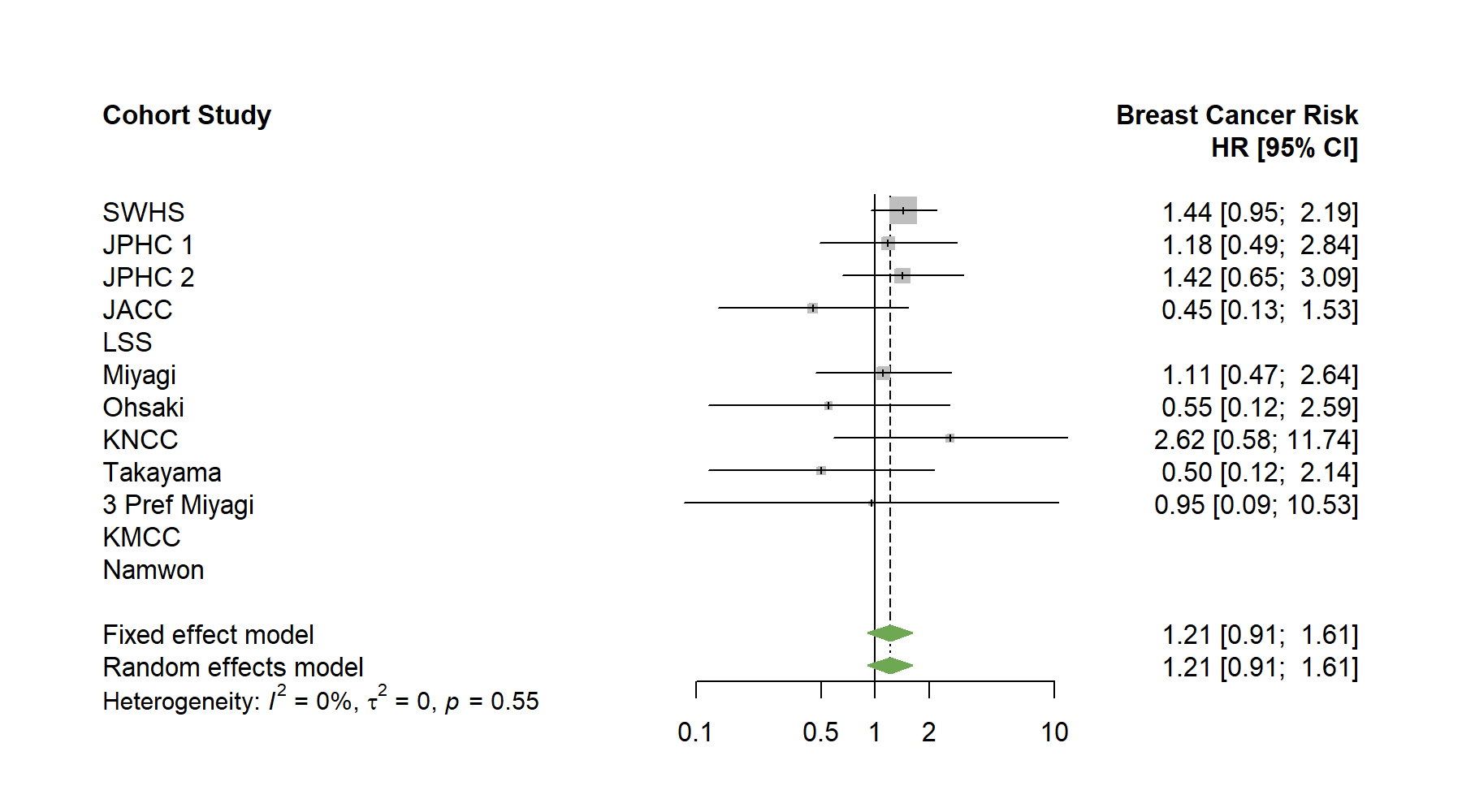  (d) | 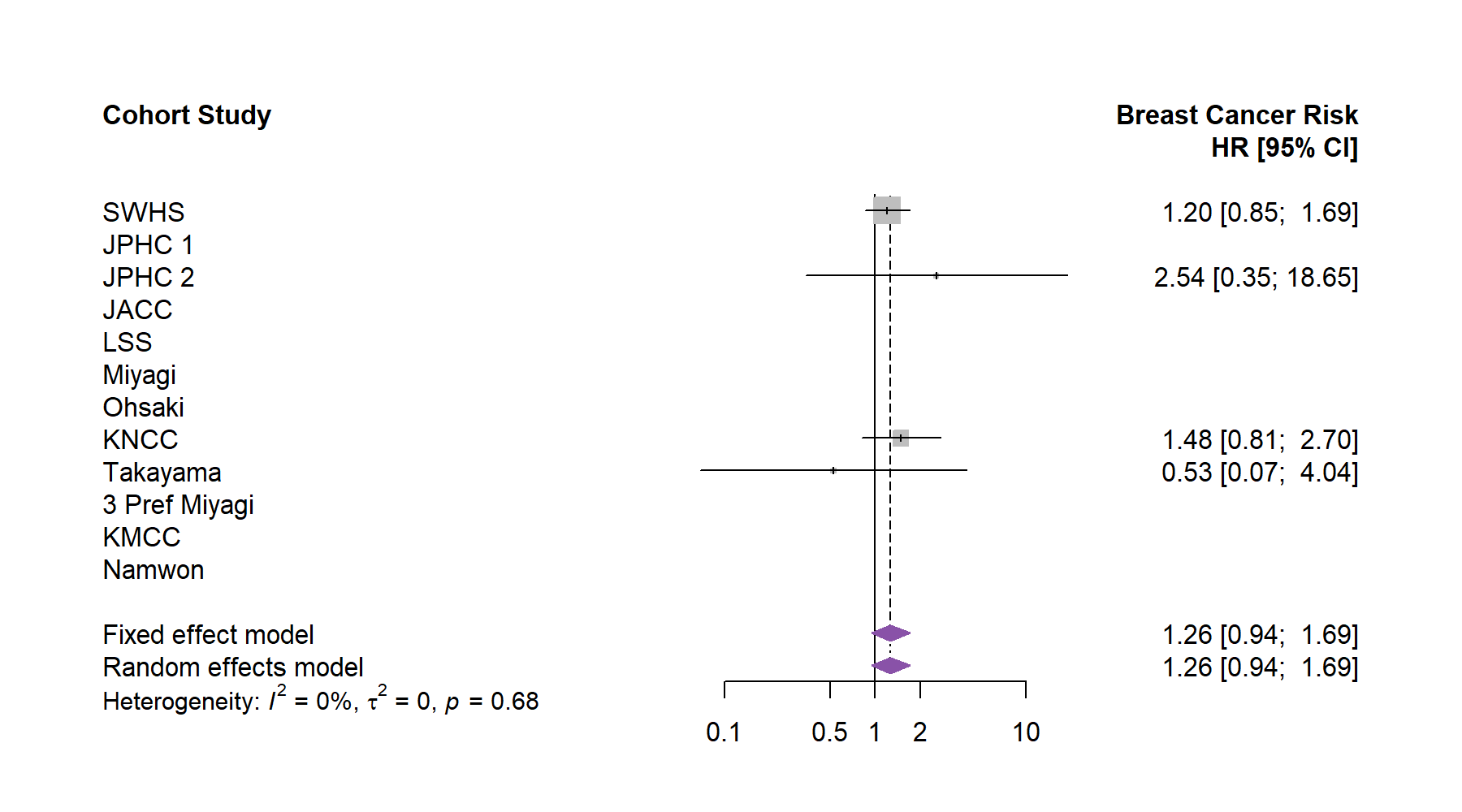  (e) |

Supplementary Figure S6. Forest plot showing the meta-analysis of hazard ratios for breast cancer risk in participants with a menarche age <13 years (versus >17 years) according to birth cohort. a: ≤1920s birth cohort; b: 1930s birth cohort; c: 1940 birth cohort; d: ≥1950s birth cohort.

| 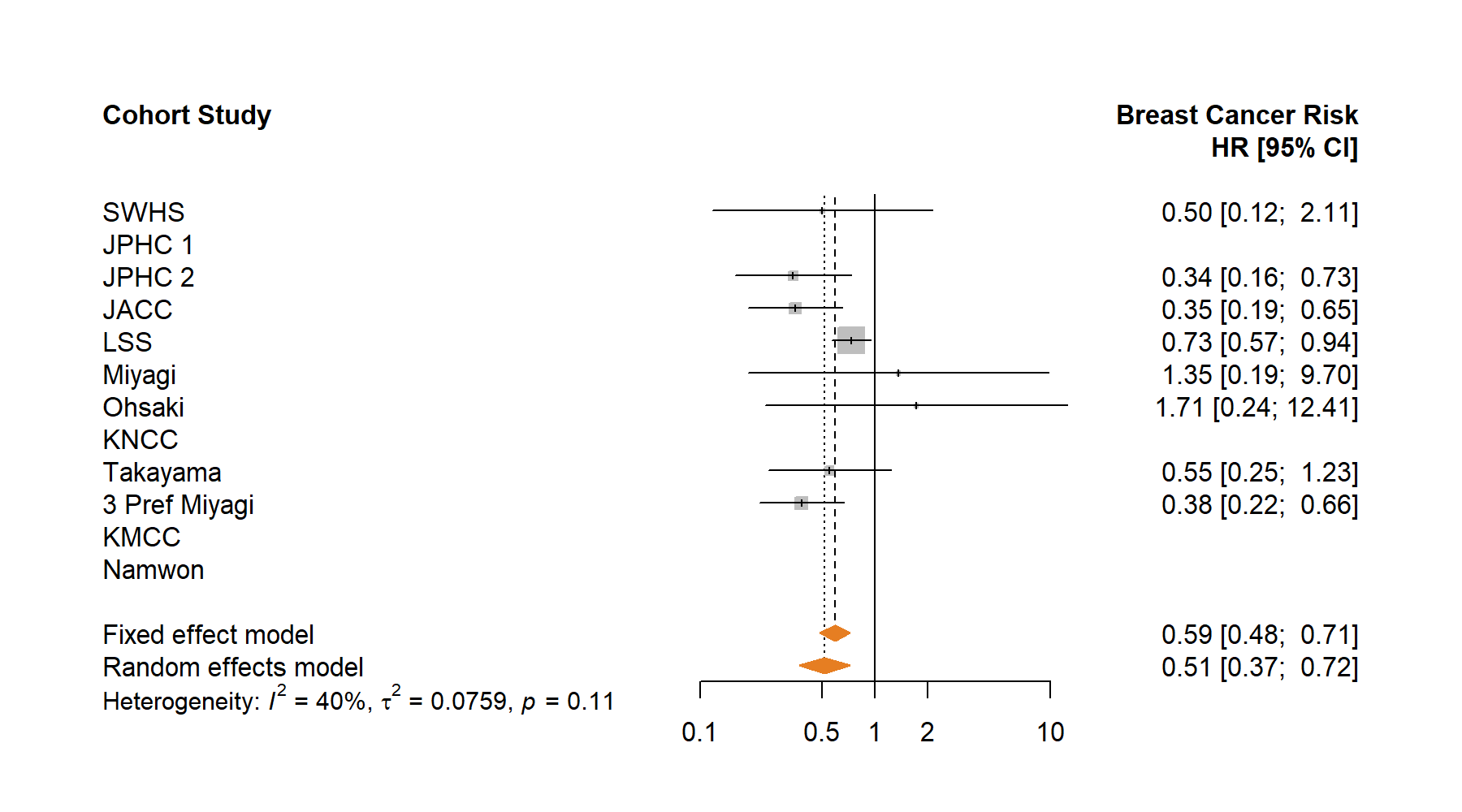  (b) | 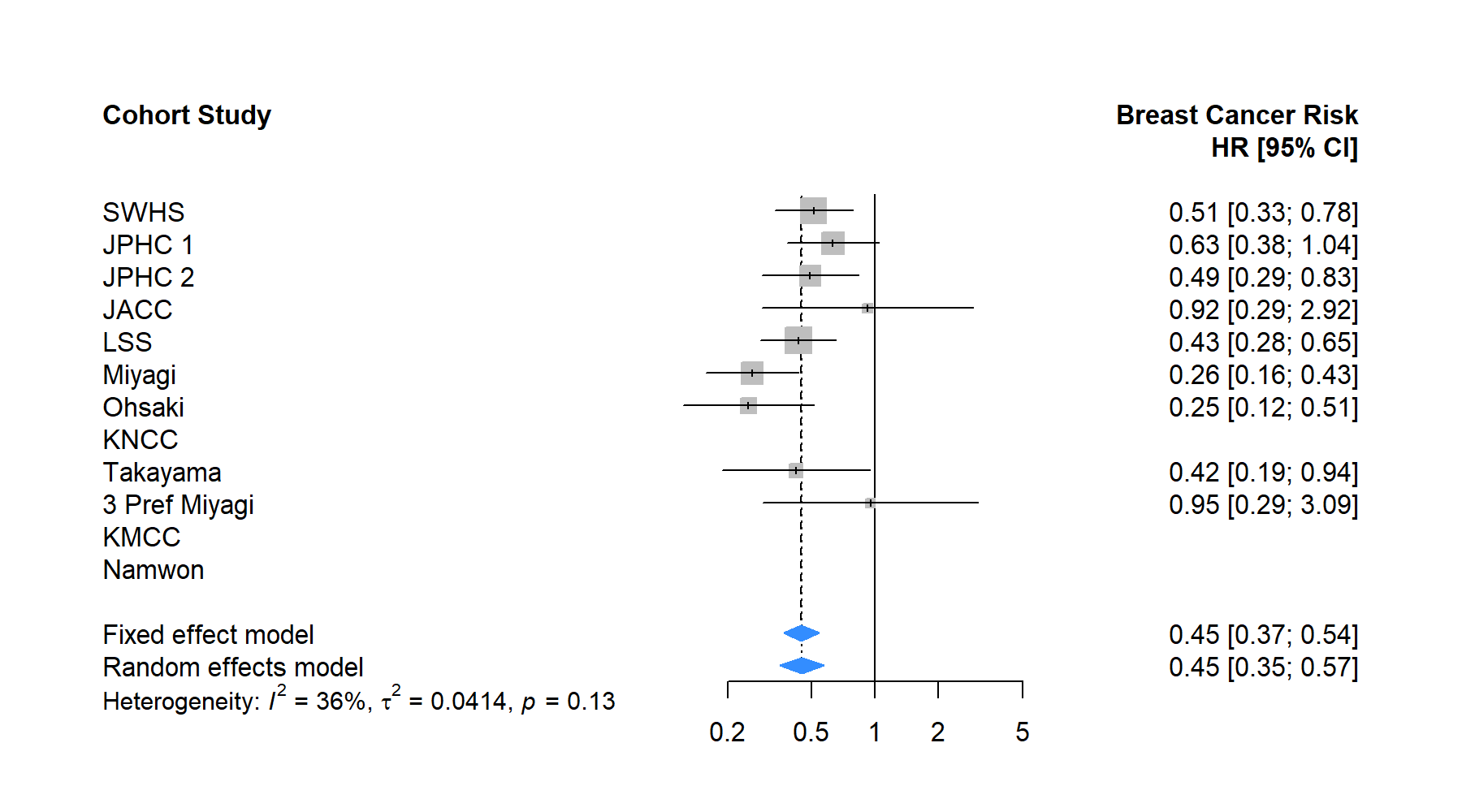  (c) |
| --- | --- |
| 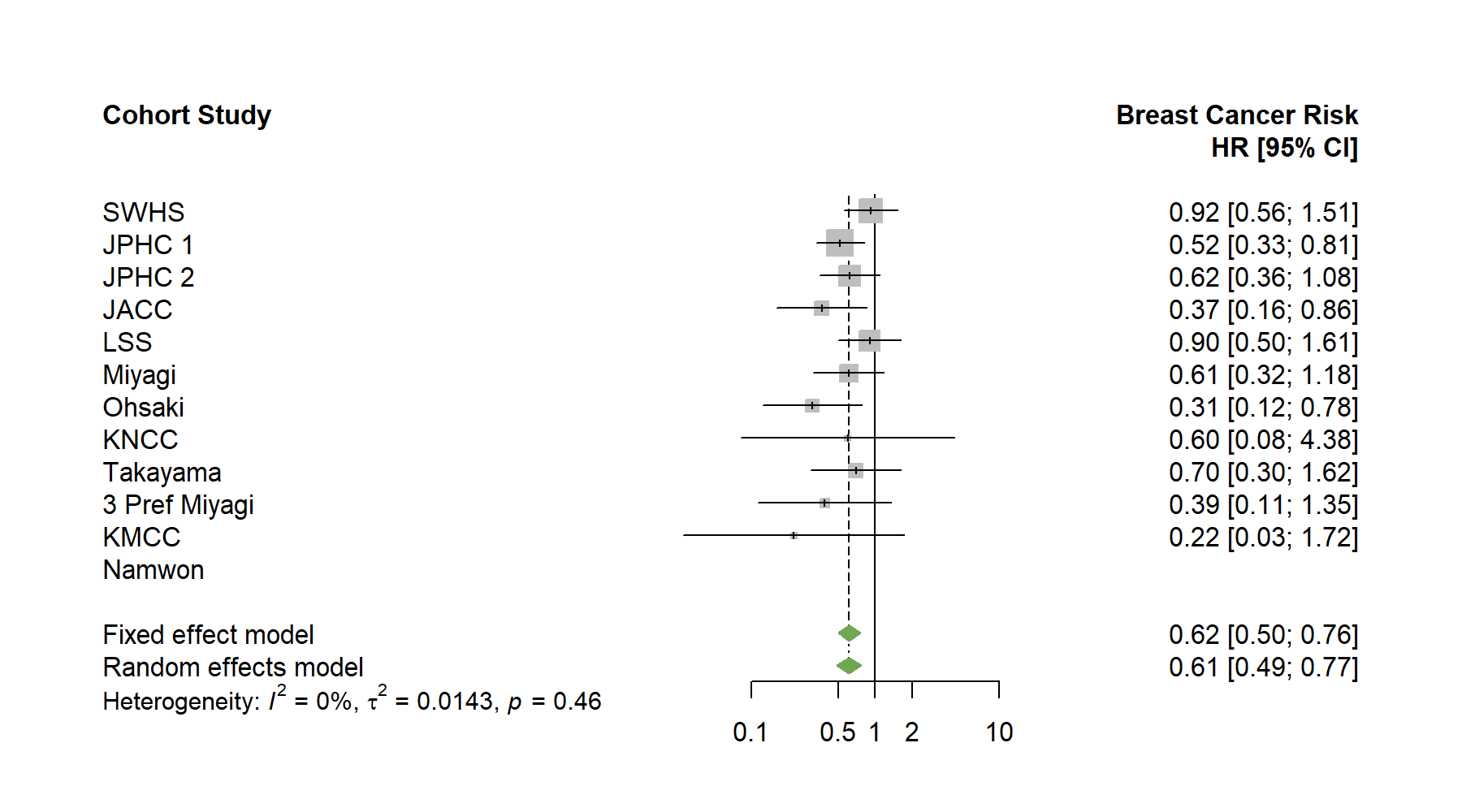  (d) | 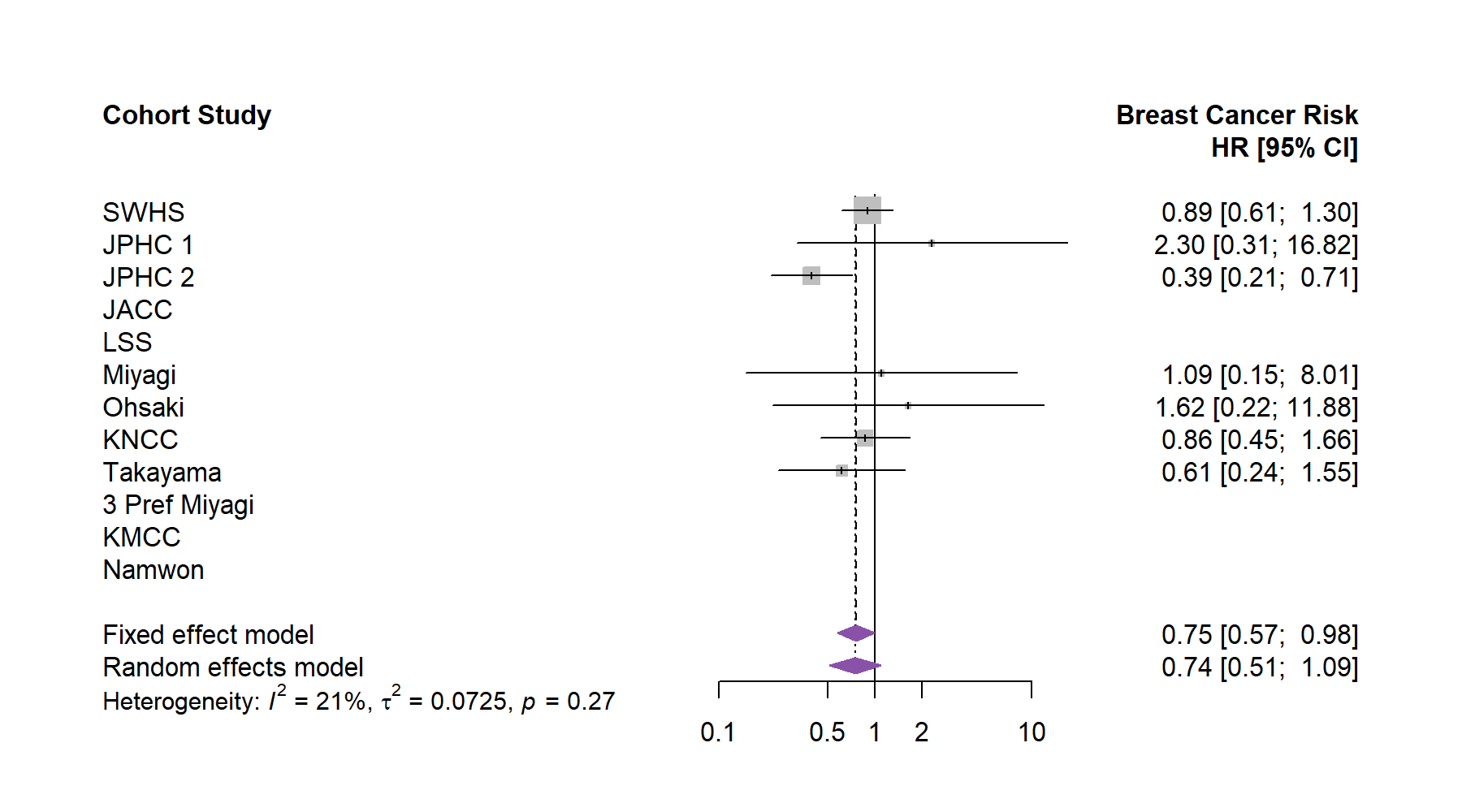  (e) |

Supplementary Figure S7. Forest plot showing the meta-analysis of hazard ratios for breast cancer risk in parous participants (versus nulliparous) according to birth cohort. a: ≤1920s birth cohort; b: 1930s birth cohort; c: 1940 birth cohort; d: ≥1950s birth cohort.

| 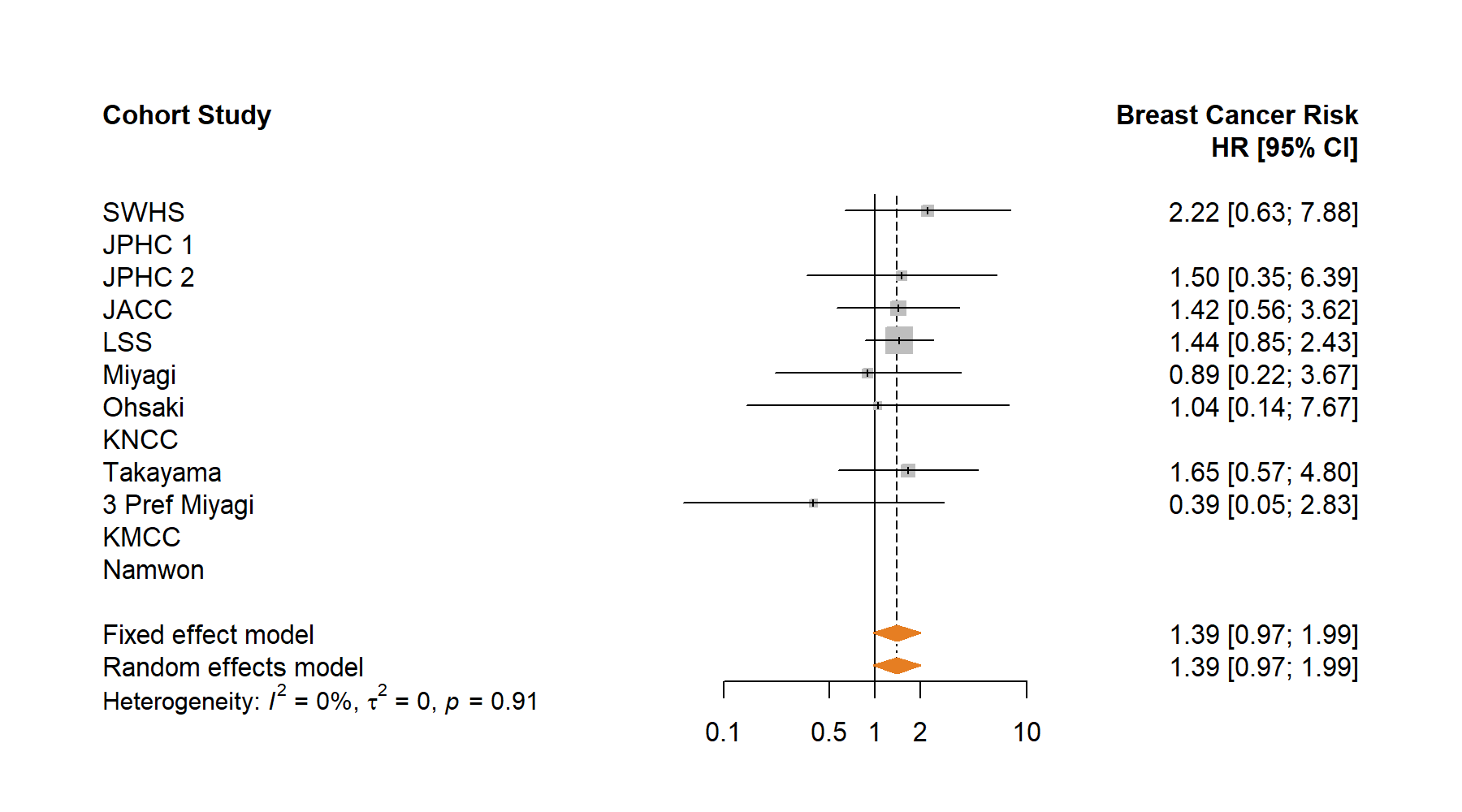  (b) | 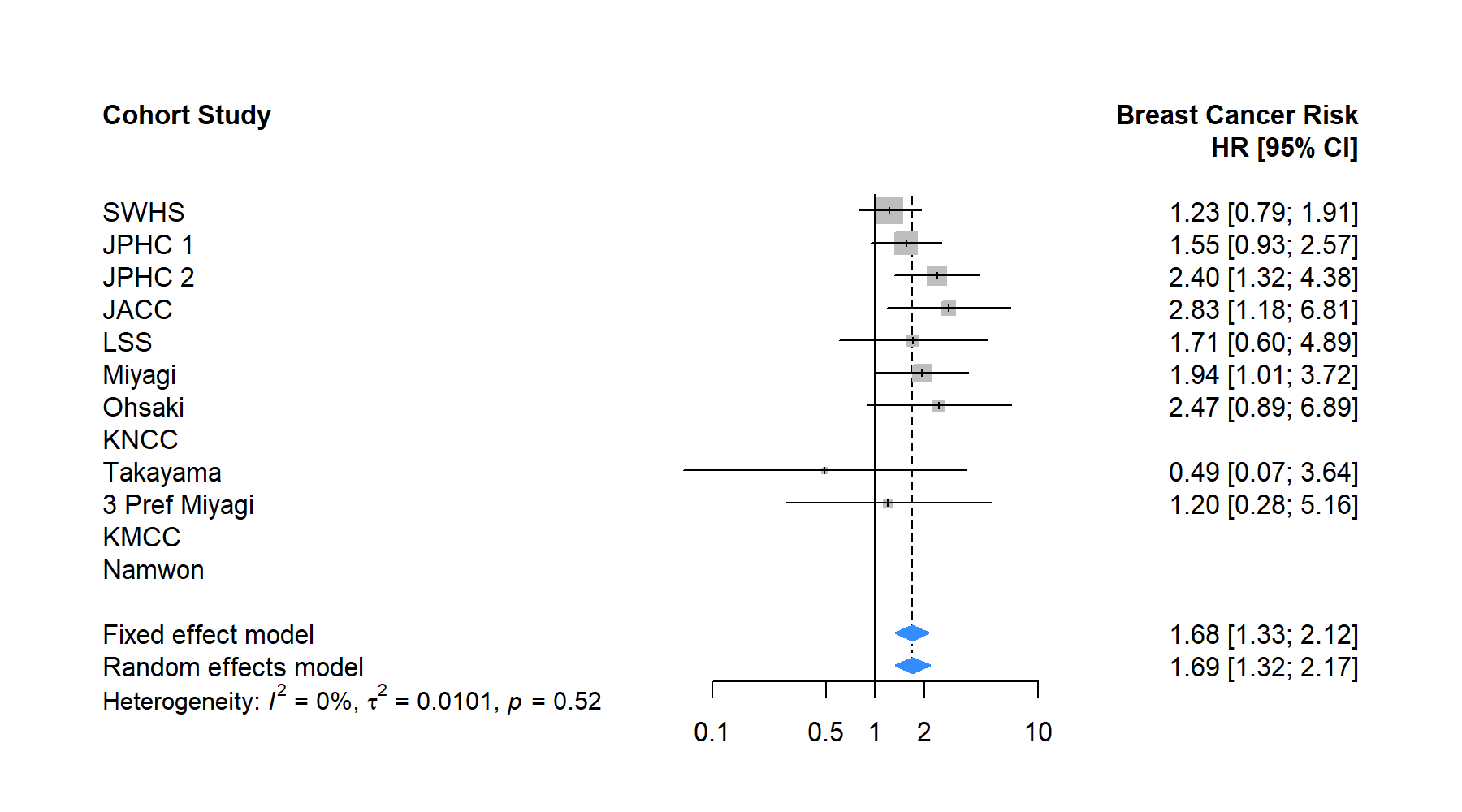  (c) |
| --- | --- |
| 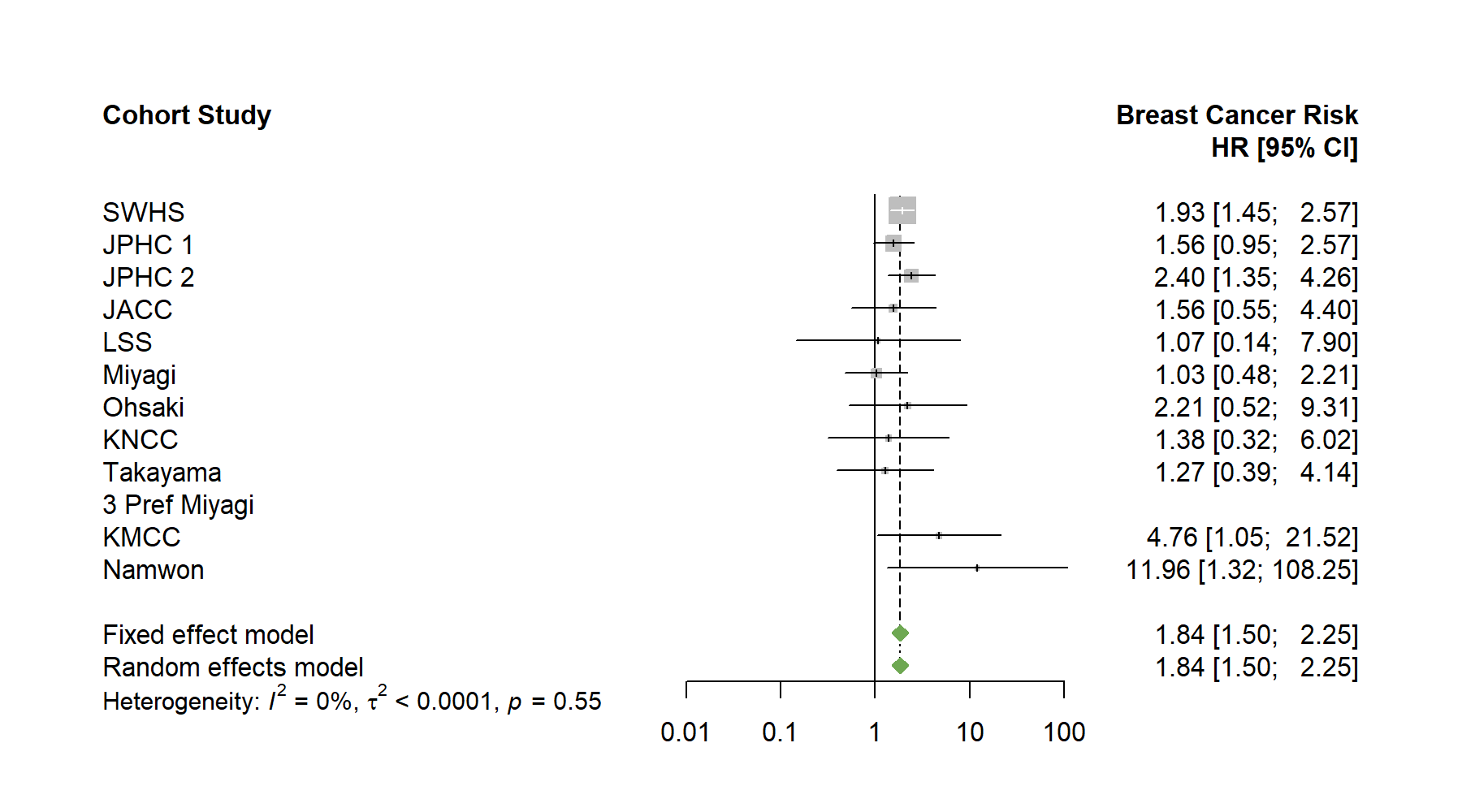  (d) | 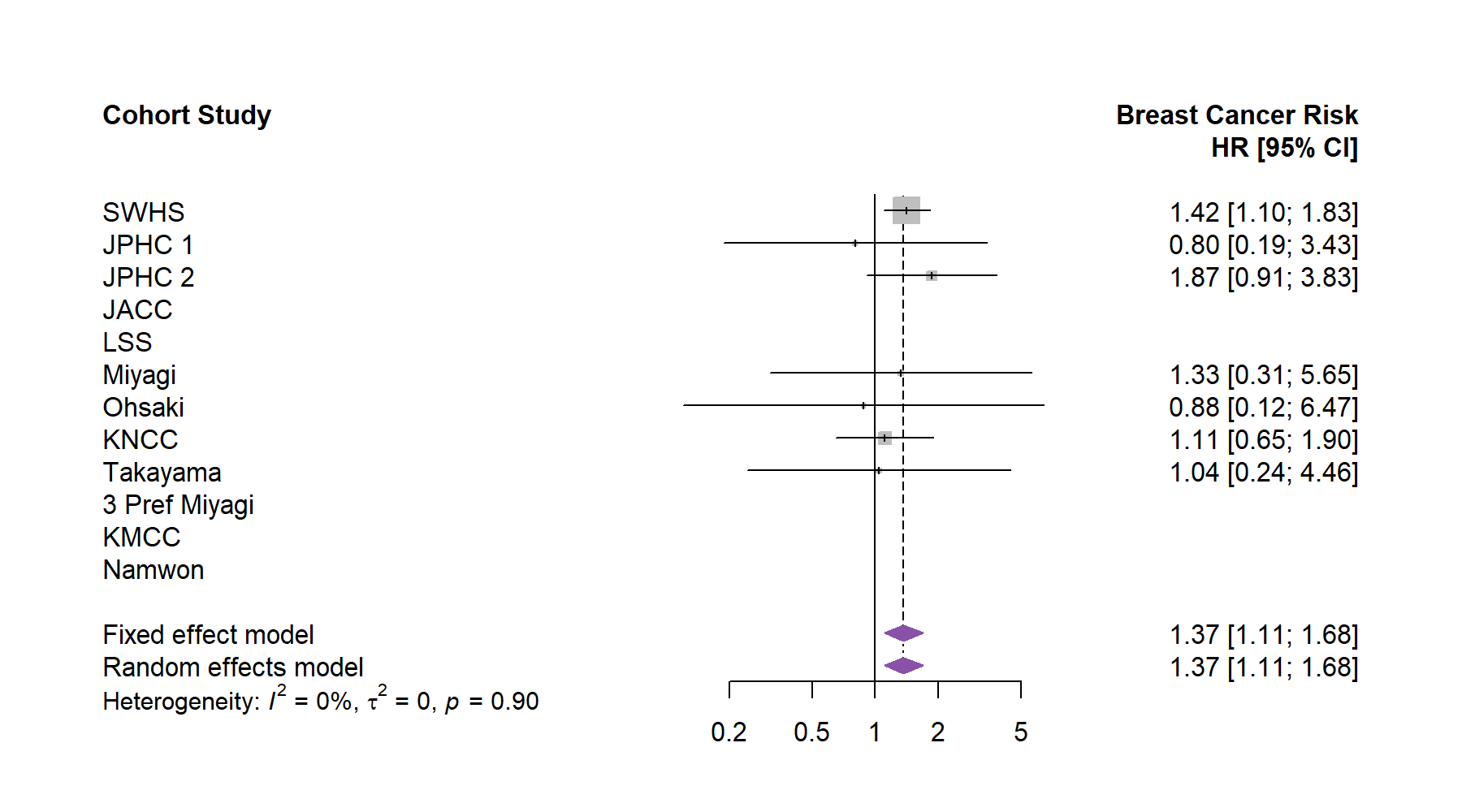  (e) |

Supplementary Figure S8. Forest plot showing the meta-analysis of hazard ratios for breast cancer risk in participants with an age at first delivery >30 years (versus 21–25 years) according to birth cohort. a: ≤1920s birth cohort; b: 1930s birth cohort; c: 1940 birth cohort; d: ≥1950s birth cohort.

| 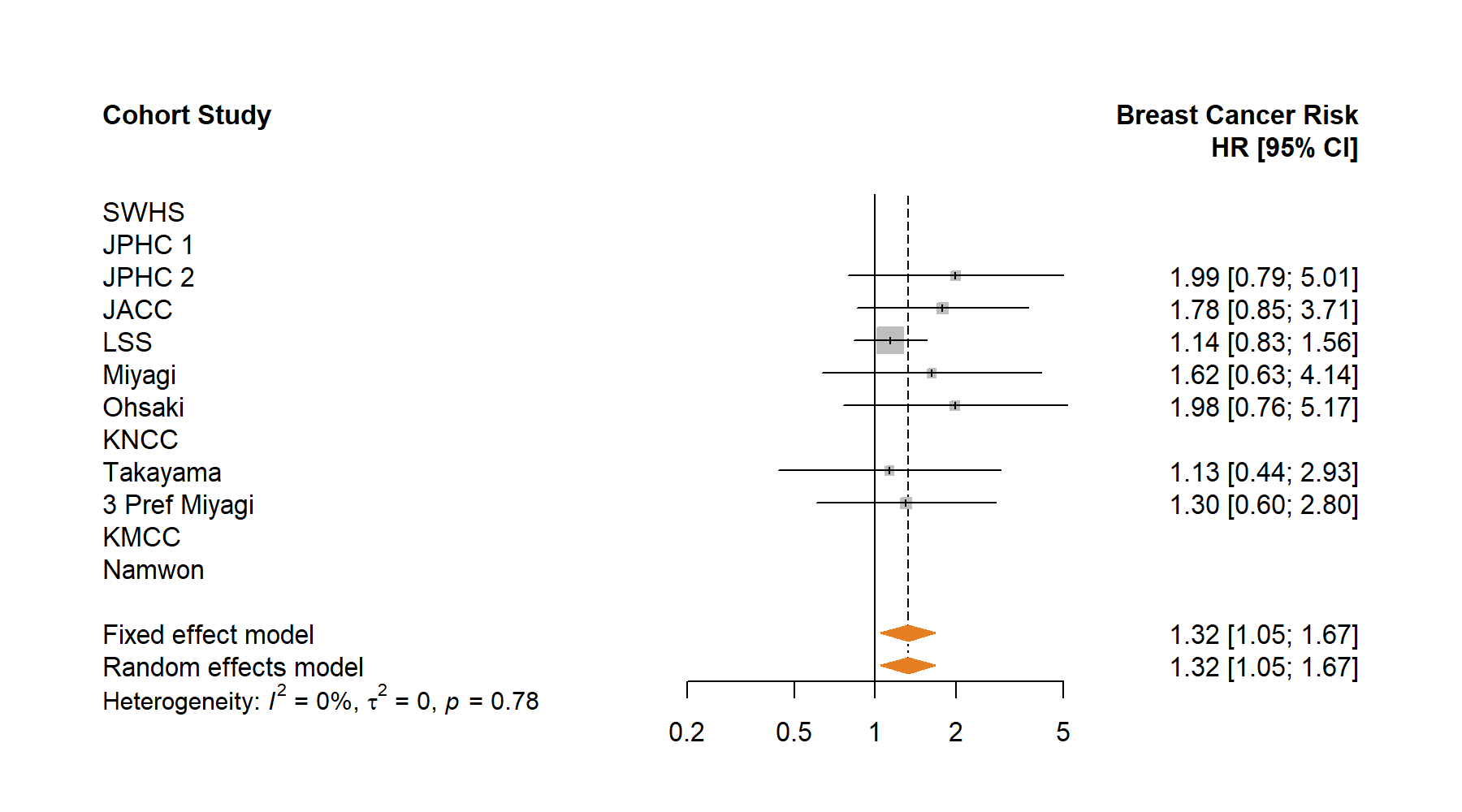  (b) | 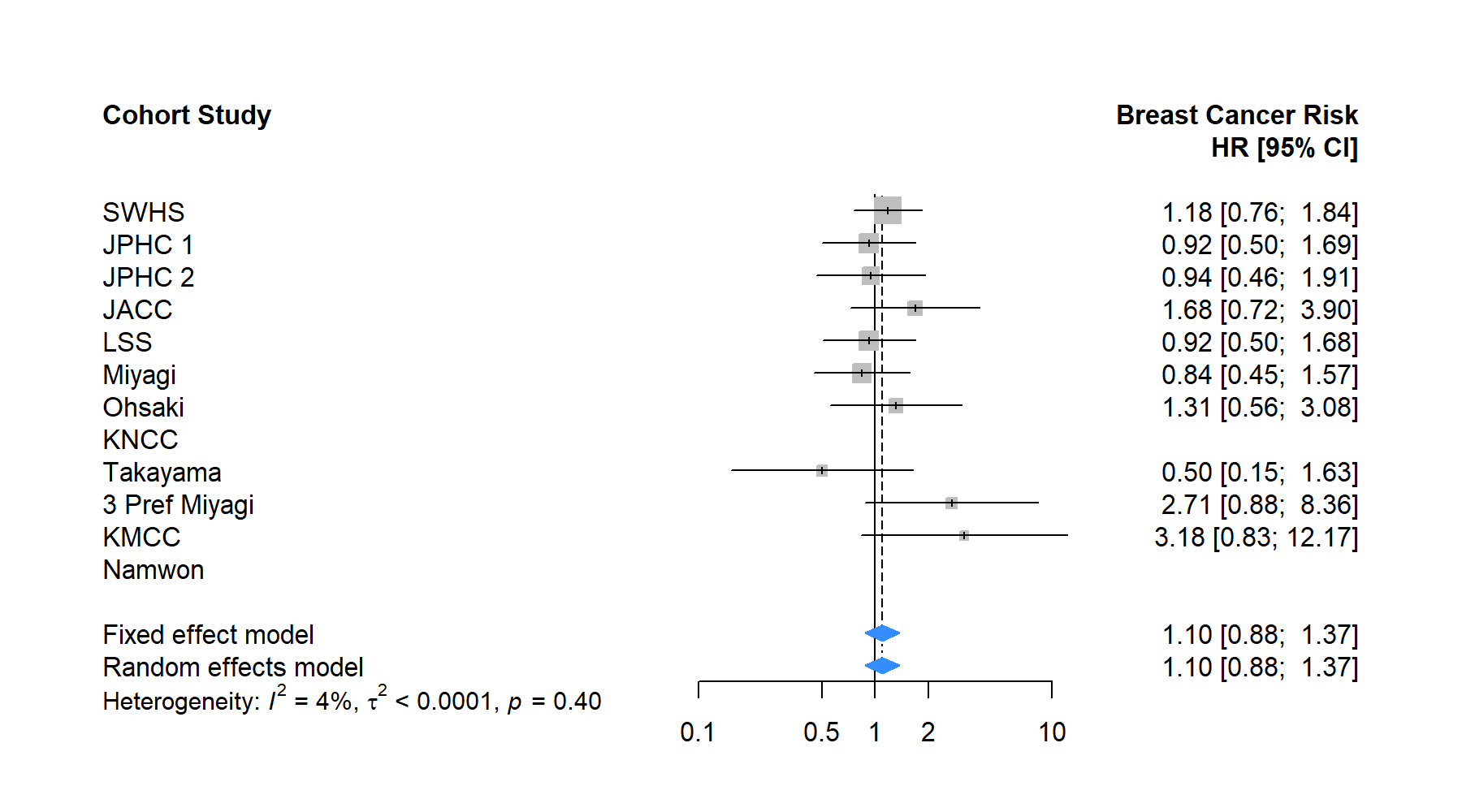  (c) |
| --- | --- |
| 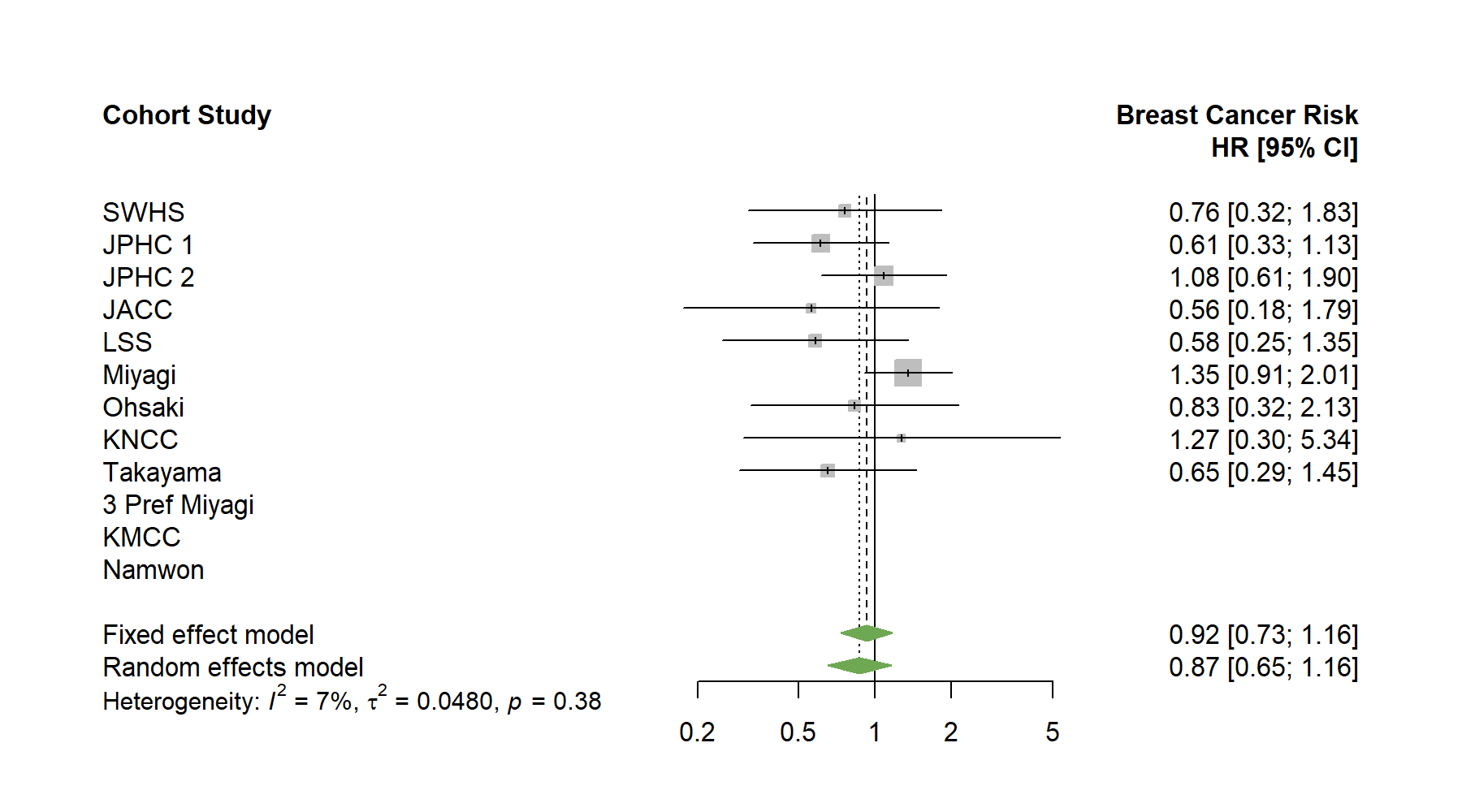  (d) | 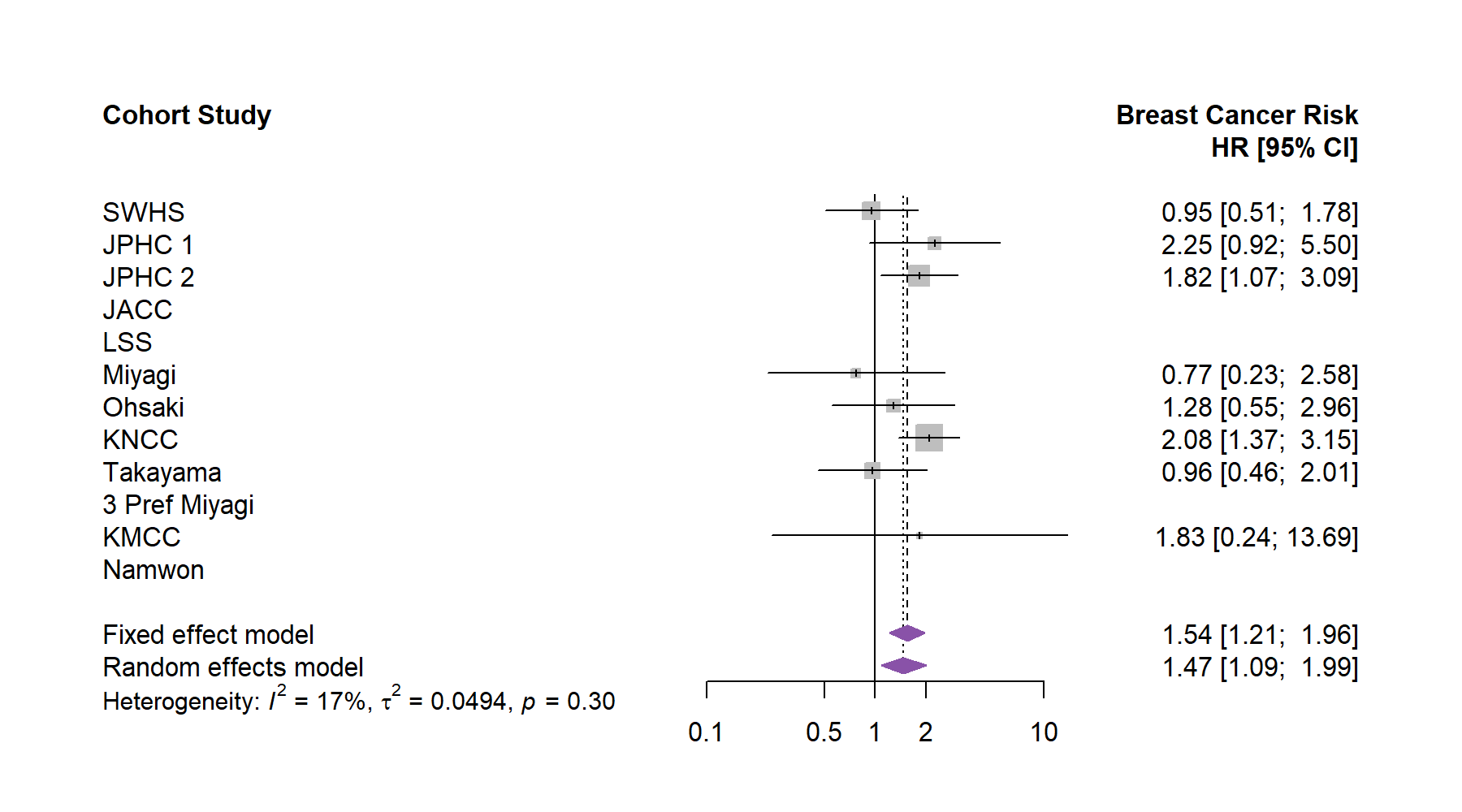  (e) |

Supplementary Figure S9. Forest plot showing the meta-analysis of hazard ratios for breast cancer risk in ever smokers (versus never smokers) according to birth cohort. a: ≤1920s birth cohort; b: 1930s birth cohort; c: 1940 birth cohort; d: ≥1950s birth cohort.

| 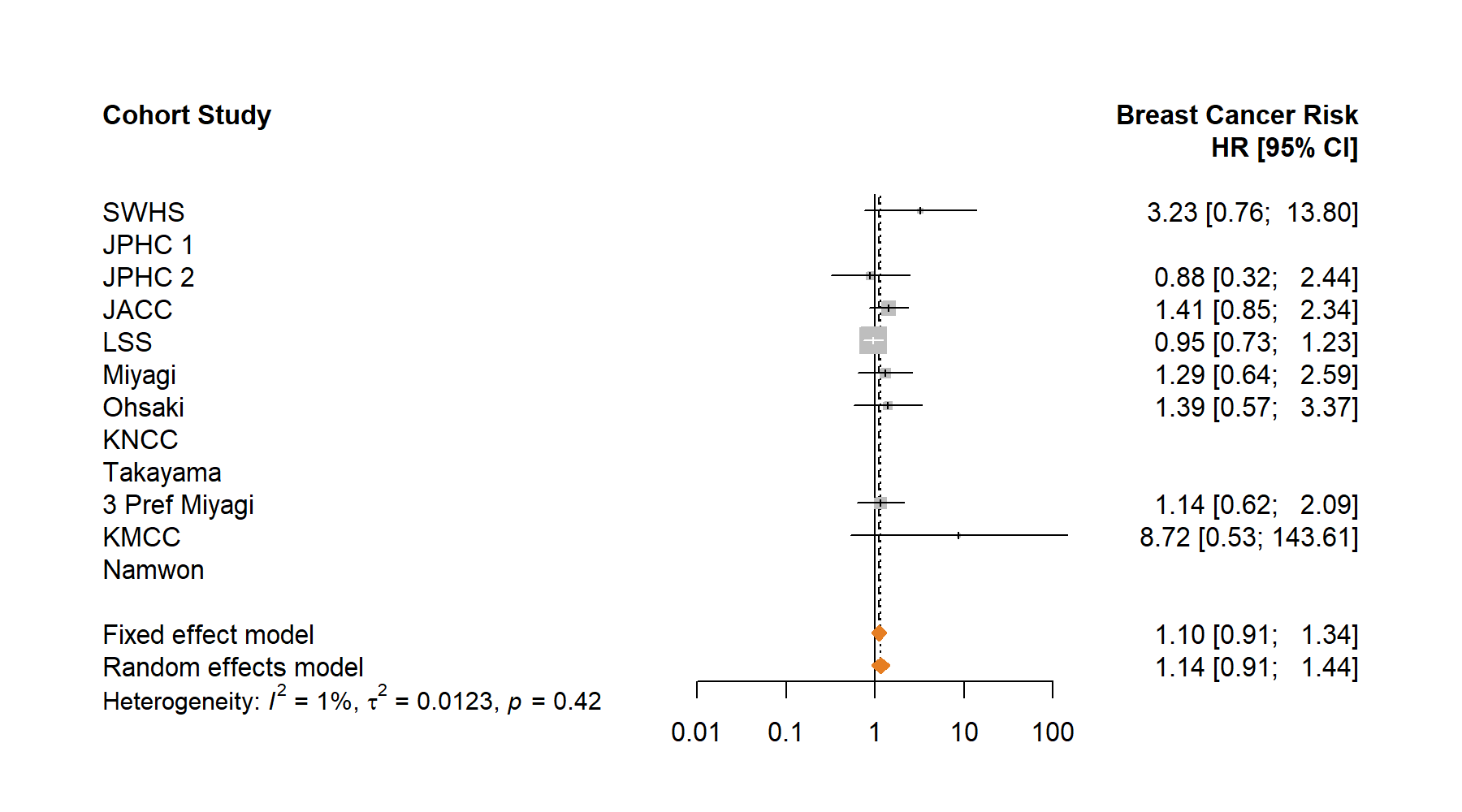  (b) | 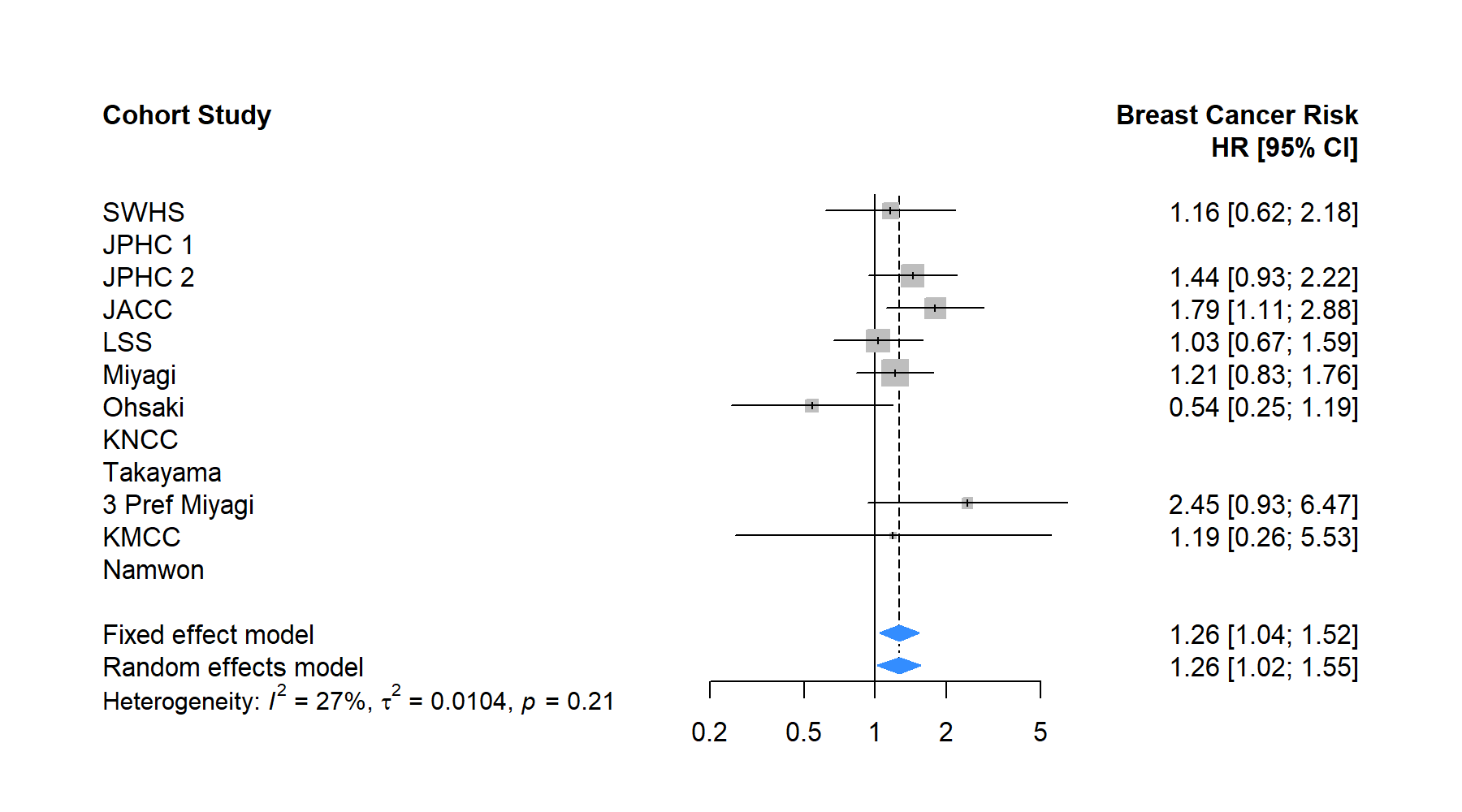  (c) |
| --- | --- |
| 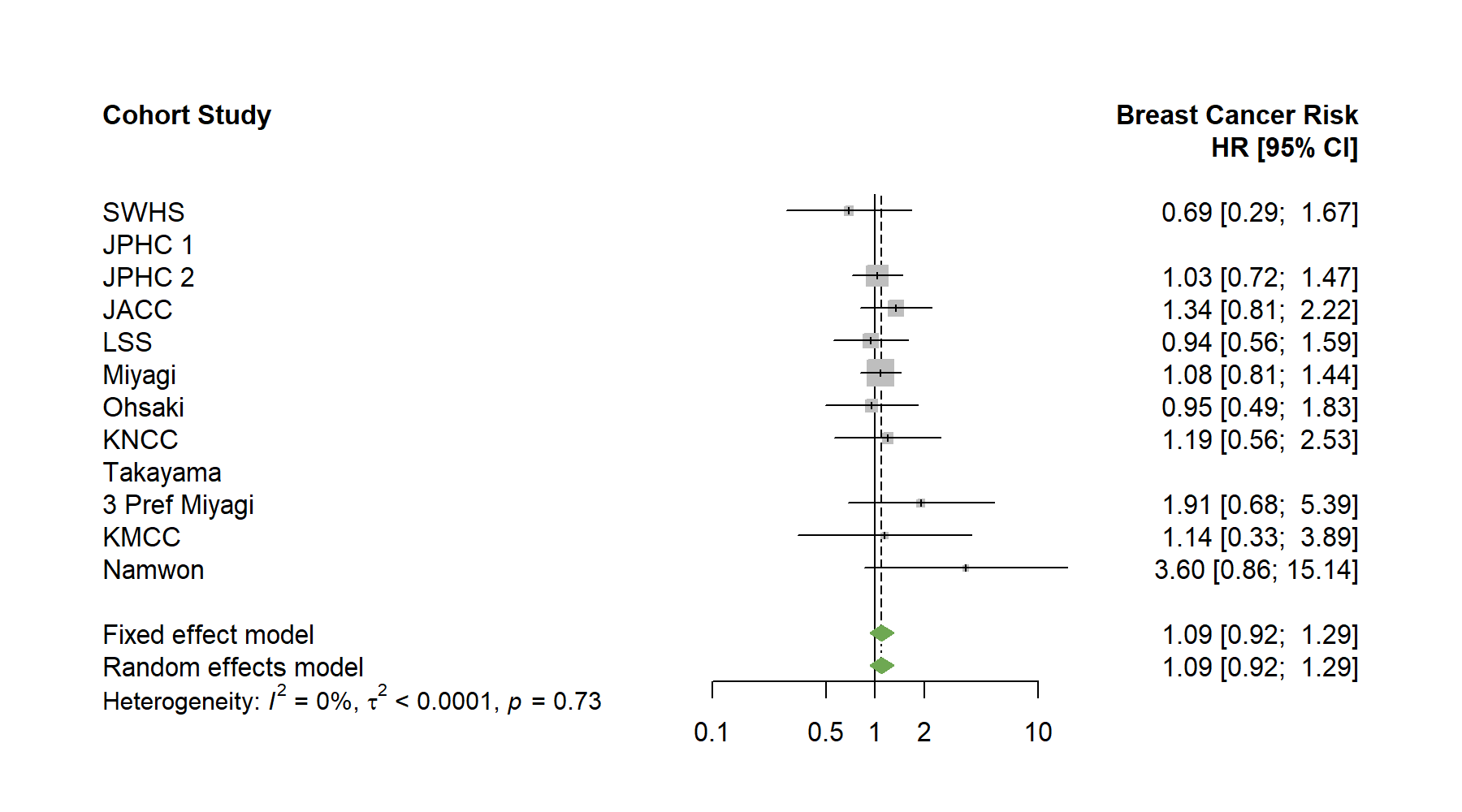  (d) | 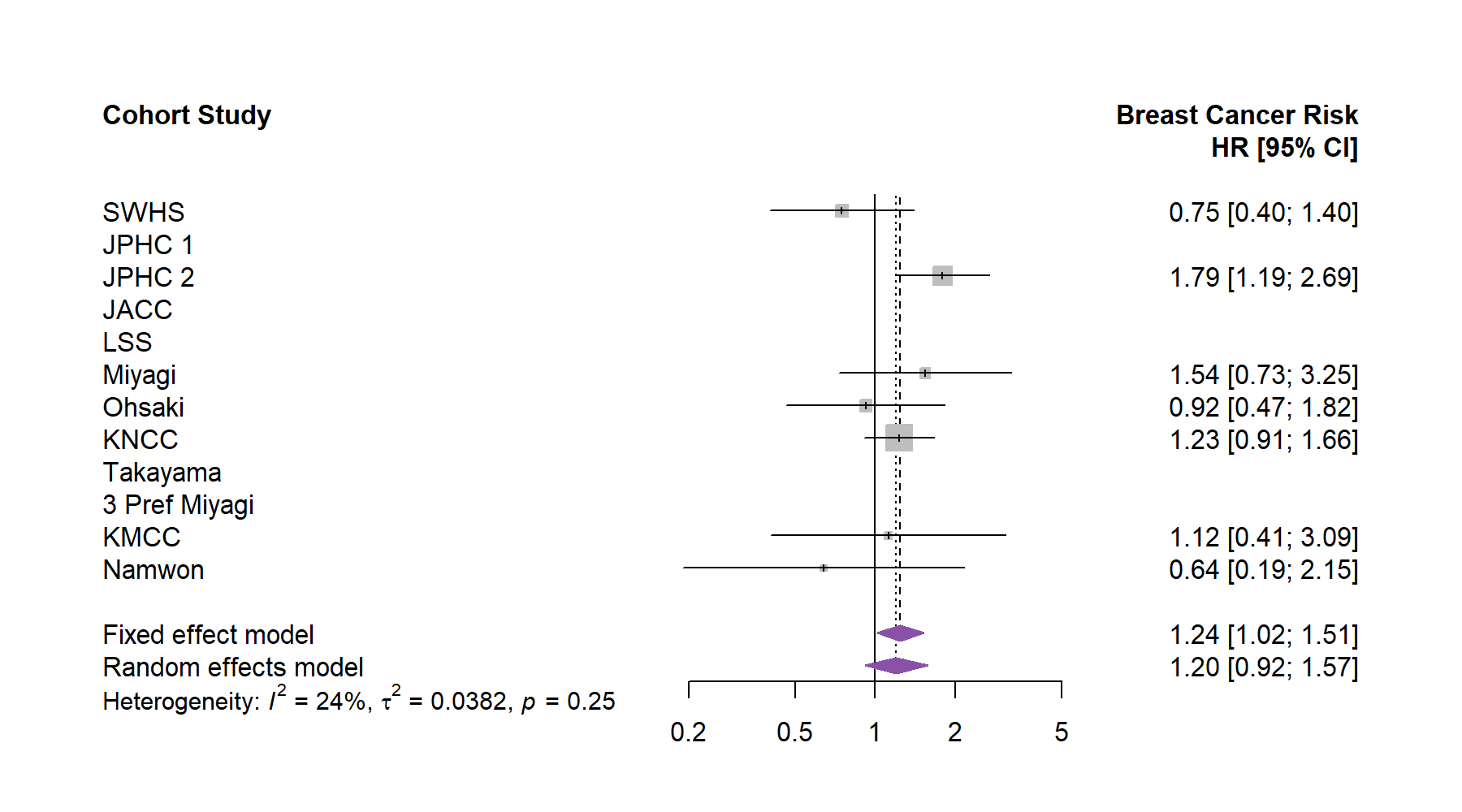  (e) |

Supplementary Figure S10. Forest plot showing the meta-analysis of hazard ratios for breast cancer risk in drinkers (versus non-drinkers) according to birth cohort. a: ≤1920s birth cohort; b: 1930s birth cohort; c: 1940 birth cohort; d: ≥1950s birth cohort.
